# Regulation of ectopic heterochromatin-mediated epigenetic diversification by the JmjC family protein Epe1

**DOI:** 10.1371/journal.pgen.1008129

**Published:** 2019-06-17

**Authors:** Masato Sorida, Takahiro Hirauchi, Hiroaki Ishizaki, Wataru Kaito, Atsushi Shimada, Chie Mori, Yuji Chikashige, Yasushi Hiraoka, Yutaka Suzuki, Yasuyuki Ohkawa, Hiroaki Kato, Shinya Takahata, Yota Murakami

**Affiliations:** 1 Graduate School of Chemical Sciences and Engineering, Hokkaido University, Sapporo, Japan; 2 Department of Chemistry, Faculty of Science, Hokkaido University, Sapporo, Japan; 3 Advanced ICT Research Institute Kobe, National Institute of Information and Communications Technology, Kobe, Japan; 4 Graduate School of Frontier Biosciences, Osaka University, Suita, Japan; 5 Department of Computational Biology and Medical Science, Graduate School of Frontier Sciences, the University of Tokyo, Kashiwa, Japan; 6 Division of Transcriptomics, Medical Institute of Bioregulation, Kyushu University, Fukuoka, Japan; 7 Department of Biochemistry, Shimane University School of Medicine, Izumo, Japan; University of Edinburgh, UNITED KINGDOM

## Abstract

H3K9 methylation (H3K9me) is a conserved marker of heterochromatin, a transcriptionally silent chromatin structure. Knowledge of the mechanisms for regulating heterochromatin distribution is limited. The fission yeast JmjC domain-containing protein Epe1 localizes to heterochromatin mainly through its interaction with Swi6, a homologue of heterochromatin protein 1 (HP1), and directs JmjC-mediated H3K9me demethylation *in vivo*. Here, we found that loss of *epe1* (*epe1*Δ) induced a red-white variegated phenotype in a red-pigment accumulation background that generated uniform red colonies. Analysis of isolated red and white colonies revealed that silencing of genes involved in pigment accumulation by stochastic ectopic heterochromatin formation led to white colony formation. In addition, genome-wide analysis of red- and white-isolated clones revealed that *epe1*Δ resulted in a heterogeneous heterochromatin distribution among clones. We found that Epe1 had an N-terminal domain distinct from its JmjC domain, which activated transcription in both fission and budding yeasts. The N-terminal transcriptional activation (NTA) domain was involved in suppression of ectopic heterochromatin-mediated red-white variegation. We introduced a single copy of Epe1 into *epe1*Δ clones harboring ectopic heterochromatin, and found that Epe1 could reduce H3K9me from ectopic heterochromatin but some of the heterochromatin persisted. This persistence was due to a latent H3K9me source embedded in ectopic heterochromatin. Epe1H297A, a canonical JmjC mutant, suppressed red-white variegation, but entirely failed to remove already-established ectopic heterochromatin, suggesting that Epe1 prevented stochastic *de novo* deposition of ectopic H3K9me in an NTA-dependent but JmjC-independent manner, while its JmjC domain mediated removal of H3K9me from established ectopic heterochromatin. Our results suggest that Epe1 not only limits the distribution of heterochromatin but also controls the balance between suppression and retention of heterochromatin-mediated epigenetic diversification.

## Author summary

Suppression of unscheduled epigenetic alterations is important for maintenance of homogeneity among clones, while emergence of epigenetic differences is also important for adaptation or differentiation. The mechanisms that balance both processes warrant further investigation. Epe1, a fission yeast JmjC domain-containing protein, is thought to be an H3K9me demethylase that targets ectopic heterochromatin via its JmjC-dependent demethylation function. Here we found that loss of *epe1* induced stochastic ectopic heterochromatin formation genome-wide, suggesting that the fission yeast genome had multiple potential heterochromatin formation sites, which were protected by Epe1. We found that Epe1 prevented deposition of ectopic H3K9me independently of its JmjC-mediated demethylation before heterochromatin establishment. By contrast, Epe1 could attack already-established ectopic heterochromatin via its JmjC domain, but demethylation was not 100% effective, which provided a basis for epigenetic variation. Together, our findings indicate that Epe1 is involved in both maintenance and alteration of heterochromatin distribution, and shed light on the mechanisms controlling individual-specific epigenome profiles.

## Introduction

Heterochromatin is a silent chromatin structure characterized by methylation of histone H3 at lysine 9 (H3K9me), to which heterochromatin protein 1 (HP1) binds and recruits various effectors including silencing factors. Euchromatin, another well-defined chromatin structure, is generally open and accessible to the transcriptional machinery. Protecting the genome from improper heterochromatin formation in euchromatin regions is important for constitutive gene expression. On the other hand, heterogeneous heterochromatin distribution, which leads to perturbation of gene expression, can contribute to adaptation to specific conditions.

The fission yeast *Schizosaccharomyces pombe* is a well-established model organism to analyze heterochromatin because of its conserved but simplified heterochromatin assembly system. H3K9 methylation is mediated by the sole H3K9 methyltransferase Clr4 [1, 2]. Constitutive heterochromatin in fission yeast is limited to centromeric repeats, subtelomeric regions, and the mating-type locus, while the other genomic regions consist almost entirely of euchromatin [3]. Centromeric heterochromatin formation depends mainly on the RNAi pathway that requires Dicer and Argonaute proteins encoded by *dcr1* and *ago1*, respectively [4]. Subtelomeric heterochromatin assembly relies redundantly on RNAi and the telomere DNA-binding protein Taz1 [5], but this assembly scheme might not be applicable to subtelomeric heterochromatin of chromosome III. Chromosome III subtelomeres contain ribosomal DNA (rDNA) repeats, which lie adjacent to telomeres and are subject to heterochromatin silencing. However, the assembly mechanism of heterochromatin at rDNA repeats is largely unknown [3, 6–8]. Heterochromatin formation at the mating-type locus depends redundantly on RNAi and ATF/CREB family proteins Atf1/Pcr1 [9].

The JmjC domain is conserved from yeast to human, and demethylates methyl-lysines of histones using Fe^2+^ and 2-oxoglutarate as cofactors [10]. A JmjC domain-containing protein, *S*. *pombe* Epe1, has been identified as an anti-silencing factor at heterochromatin-euchromatin boundaries [11–14]. The Cul4-Ddb1^Cdt2^ ubiquitin ligase complex moderately degrades Epe1 located inside the boundaries [15, 16]. Overexpression of Epe1 reduces centromeric di-methylated H3K9 (H3K9me2) *in vivo* [12, 17] and facilitates transcription in heterochromatin and at specific euchromatin loci [12, 13, 17]. In addition, Epe1 promotes histone turnover in heterochromatin [18]. Epe1 physically interacts with the HP1 homologs Swi6 and Chp2 and localizes to heterochromatin in a largely Swi6-dependent manner [12, 15, 19, 20]. Loss of *epe1* partially destabilizes centromeric heterochromatin, whereas combined loss of *epe1* and *dcr1* or *ago1* retains an *epe1*Δ-like silenced state at centromeres, indicating that *epe1*Δ bypasses the requirement for RNAi [12, 13, 21]. The mechanisms underlying these phenotypes remain unknown. Despite accumulating *in vivo* evidence, Epe1 does not display demethylation activity *in vitro* [22]. Epe1 may therefore not be a canonical H3K9 demethylase.

Recent studies suggest genome-wide functions of Epe1. Twenty-one small H3K9me peaks, designated heterochromatin islands (*Is*), exist in euchromatic regions and Epe1 represses expansion of the islands as well as emergence of additional islands [23]. Combined loss of Epe1 and the histone acetyltransferase Mst2 induces strong ectopic heterochromatin on many euchromatic loci including essential genes, resulting in a severe growth defect, which is eliminated by heterochromatin formation on heterochromatin assembly genes [24]. An artificial Clr4-tethering system establishes extensive heterochromatin on euchromatic regions in the presence of Epe1, and after release of tethered Clr4, the heterochromatin is disrupted by Epe1 in a JmjC-dependent manner [25, 26]. These studies suggest that Epe1 targets ectopic heterochromatin and antagonizes heterochromatin formation in euchromatic regions via its JmjC-dependent function, although whether Epe1 actually removes H3K9me in spontaneously established ectopic heterochromatin is not known.

Here, we found that loss of Epe1 resulted in the repression of a ribonucleotide synthesis gene and a change in cell phenotype by mediating ectopic heterochromatin formation on this gene. Further analyses showed that ectopic heterochromatin was stochastically established, thereby producing an epigenetically diversified population of *epe1*Δ clone cells. Surprisingly, Epe1 prevented ectopic H3K9me deposition independently of both its JmjC-mediated demethylation and heterochromatin association ability. We identified the N-terminal transcriptional activation (NTA) domain of Epe1 and it contributed to the prevention function. Epe1 removed H3K9me from already-established ectopic heterochromatin, but the removal was not complete. The persistent ectopic heterochromatin harbored an H3K9me supply source that did not deposit H3K9me in the presence of Epe1 (designated as latent H3K9me source). Collectively, the results show that Epe1 has two distinct functions: the protection of euchromatic regions from stochastic *de novo* ectopic heterochromatin formation via a mechanism involving its N-terminal transcriptional activation (NTA) domain, and incomplete disruption of already-established ectopic heterochromatin via its JmjC domain. Thus, Epe1 could be a key regulator in the formation of individual-specific H3K9me landscapes.

## Results

### Loss of *epe1* induces ectopic heterochromatin formation and phenotypic alterations

**Fig 1:**
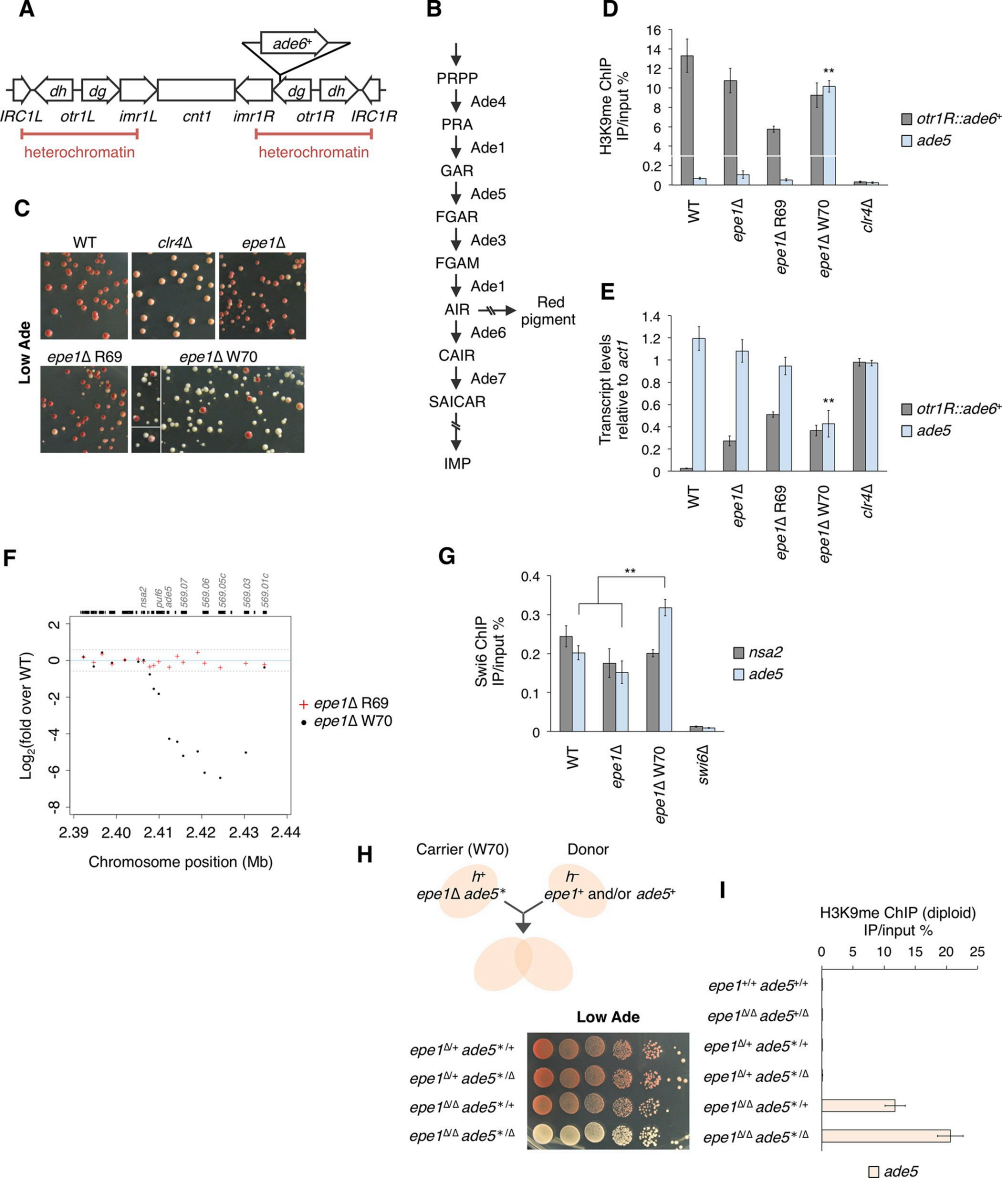
Loss of *epe1* induces ectopic heterochromatin formation and phenotypic alterations. (A) Schematic diagram showing the structure of the centromere of chromosome I (*cen1*). The position of the *ade6*^+^ marker is indicated (*otr1R*::*ade6*^+^). Endogenous *ade6* is disrupted by a loss-of-function truncation mutation (*ade6-DN/N*). (B) Biosynthetic pathway for inositol monophosphate (IMP). Ade1 is a bifunctional enzyme. Little is known about the pathway of red pigment synthesis. (C) Colony color of *epe1*Δ clones and wild-type (WT) and *clr4*Δ strains on adenine-limited (Low Ade) medium. (D) Chromatin immunoprecipitation (ChIP)-qPCR analysis of H3K9me levels at *otr1R*::*ade6*^+^ and *ade5*. **p < 0.05. *p*-values were determined using a two-tailed Student’s *t*-test comparing the indicated sample value with the value of other samples. (E) Reverse transcription and quantitative polymerase chain reaction (qRT-PCR) analysis of *otr1R*::*ade6*^+^ and *ade5* transcript levels relative to those of *act1*. **p < 0.05. *p*-values were determined using a two-tailed Student’s *t*-test comparing the indicated sample value with the value of other samples. (F) Transcriptome microarray analysis. The x-axis indicates the chromosome position of *subtel3R*. The y-axis indicates log_2_ fold changes of gene expression levels in R69 or W70 clone cells over those in WT cells. The upper panel indicates the gene positions and names corresponding to array positions. Unreliable signals with low intensity were excluded. Red cross, *epe1*Δ R69/WT; black dot, *epe1*Δ W70/WT; sky-blue solid line, y = 0; gray broken line, y = ±log_2_(1.5). (G) ChIP-qPCR analyses of Swi6 at *subtel3R* genes, *nsa2* and *ade5*. **p < 0.05 (two-tailed Student’s *t*-test). Note that the background level in Swi6 ChIP analysis was high. (H) Ten-fold serial dilution assay for diploid strains. Schematic representation of the diploid complementation system is indicated. The *epe1*Δ W70 strain was used as a carrier strain. (I) ChIP-qPCR analysis of H3K9me at *ade5* in diploids. *ade5*^+/+^ and ade5^*/+^ samples provided biallelic signals. *ade5*^+/Δ^ and ade5^*/Δ^ samples provided monoallelic signals. ChIP-qPCR and qRT-PCR data are represented as mean ± standard deviation (SD) of three independent experiments (n = 3).

An *ade6*^+^ marker inserted in a centromeric heterochromatin region (*otr1R*::*ade6*^+^; Fig 1A) is conventionally used to monitor the state of heterochromatin [27, 28]. On adenine-limited medium, silenced *otr1R*::*ade6*^+^ confers a red color on cells whereas its partial or strong desilencing confers a pink or white color, respectively, since *ade6*^+^ encodes an enzyme metabolizing 5-aminoimidazole ribotide (AIR), which develops into a red pigment through multiple steps (Fig 1B). *epe1*Δ induces heterogeneous expression of *otr1R*::*ade6*^+^ to generate a mixture of red and white colonies. Replating red colonies induces *epe1*Δ-like variegation, whereas replating white colonies frequently produces white colonies with some red/pink ones [13]. In this study, sequential white-colony isolation of an *otr1R*::*ade6*^+^ *epe1*Δ strain established several white clones (W70, W164–166) whose color was different from the light pink color of the *clr4*Δ strain, a canonical heterochromatin-defective mutant (Fig 1C, S1A and S1B Fig). W70 cells stably produced completely white colonies with a frequency of 89%.

ChIP analysis revealed that, unlike *clr4*Δ cells, *epe1*Δ W70 cells retained a substantial amount of H3K9me on *otr1R*::*ade6*^+^ (Fig 1D). *epe1*Δ W70 cells also showed low level of expression of *otr1R*::*ade6*^+^, which was comparable to that of parental *epe1*Δ cells and red-isolated *epe1*Δ R69 cells (Fig 1E). By contrast, H3K9me levels on *otr1R*::*ade6*^+^ in W164–166 cells were less than a quarter of those in the WT (S1C Fig), which probably explained the expression of *otr1R*::*ade6*^+^ and the white phenotypes. These results suggested that the white phenotype of W70 was not linked to *otr1R*::*ade6*^+^. The re-appearance of red colonies from *epe1*Δ W70 cells (Fig 1C) suggested that the white phenotype was due to epigenetic rather than genetic alterations.

To explore the cause of the white phenotype of W70, we performed microarray transcriptome analysis in W70 and R69 cells, which uncovered a cluster of genes silenced only in W70 (Fig 1F, S1D Fig). This cluster encompassed a 23 kb euchromatic region neighboring the right subtelomere of chromosome III (*subtel3R*); this region contained the *ade5* gene, whose product acts upstream of Ade6 in IMP biosynthesis (Fig 1B). Since loss of Epe1 increases H3K9me levels at *subtel1L* and *2L* [23, 24], we hypothesized that the *ade5* gene was silenced by ectopically deposited H3K9me, which arrested red pigment formation. ChIP-qPCR analysis in W70 cells revealed the presence of strong ectopic heterochromatin on *ade5* with H3K9me levels comparable to those on centromeric *ade6*^+^ in WT cells, but such ectopic heterochromatin was not detected in parental *epe1*Δ, *epe1*Δ R69 or W164–166 cells (Fig 1D, S1C Fig). Consistently, qRT-PCR analysis revealed that W70 cells had lower *ade5* expression than WT, parental *epe1*Δ, and R69 cells (Fig 1E). In W70 cells, *ade5*-like accumulation of H3K9me was observed at neighboring *puf6* and *SPCC569*.*03* but not at *nsa2*, which lay outside the cluster identified by transcriptome analysis (S1E Fig). Accordingly, we detected accumulation of Swi6 at these *subtel3R* genes but not at *nsa2* in W70 cells (Fig 1G, S1F Fig). These results suggest that silencing of the *ade5* gene by strong ectopic heterochromatin formation caused the white phenotype of W70.

To confirm the cause of the white phenotype, we performed genetic complementation of the W70 strain. We supplied single copies of *epe1*^+^ and/or *ade5*^+^ using a diploid complementation system (Fig 1H). Control diploid strains displayed expected phenotypes: *epe1*^+/+^ and *epe1*^Δ/+^, red; *epe1*^Δ/Δ^, red-white variegation; and *ade5*^Δ/Δ^, completely white (S1G Fig). We designated the ectopic heterochromatin-containing allele in W70 as ade5*. When the W70 strain (*epe1*Δ ade5*) was mated with an *epe1*Δ *ade5*Δ strain to generate *epe1*^Δ/Δ^ ade5^*/Δ^, the white phenotype of *epe1*Δ ade5* was retained, indicating that no alleles except *epe1*Δ and ade5* of W70 caused the white phenotype (Fig 1H). By contrast, introduction of *ade5*^*+*^ complemented the white phenotype (*epe1*^Δ/Δ^ ade5^*/+^), showing that ade5* was responsible for the white phenotype. Similarly, provision of *epe1*^+^ complemented the white phenotype of *epe1*Δ ade5* (*epe1*^Δ/+^ ade5^*/Δ^). ChIP analysis of these diploid cells showed the loss of H3K9me at the *ade5* region, indicating that re-introduction of Epe1 promoted demethylation of H3K9me at ade5* (Fig 1I). Thus, we concluded that ectopic heterochromatin-mediated repression of *ade5* caused the white phenotype of the *epe1*Δ W70 strain.

### The red-white variegation phenotype is linked to stochastic ectopic heterochromatin formation

**Fig 2:**
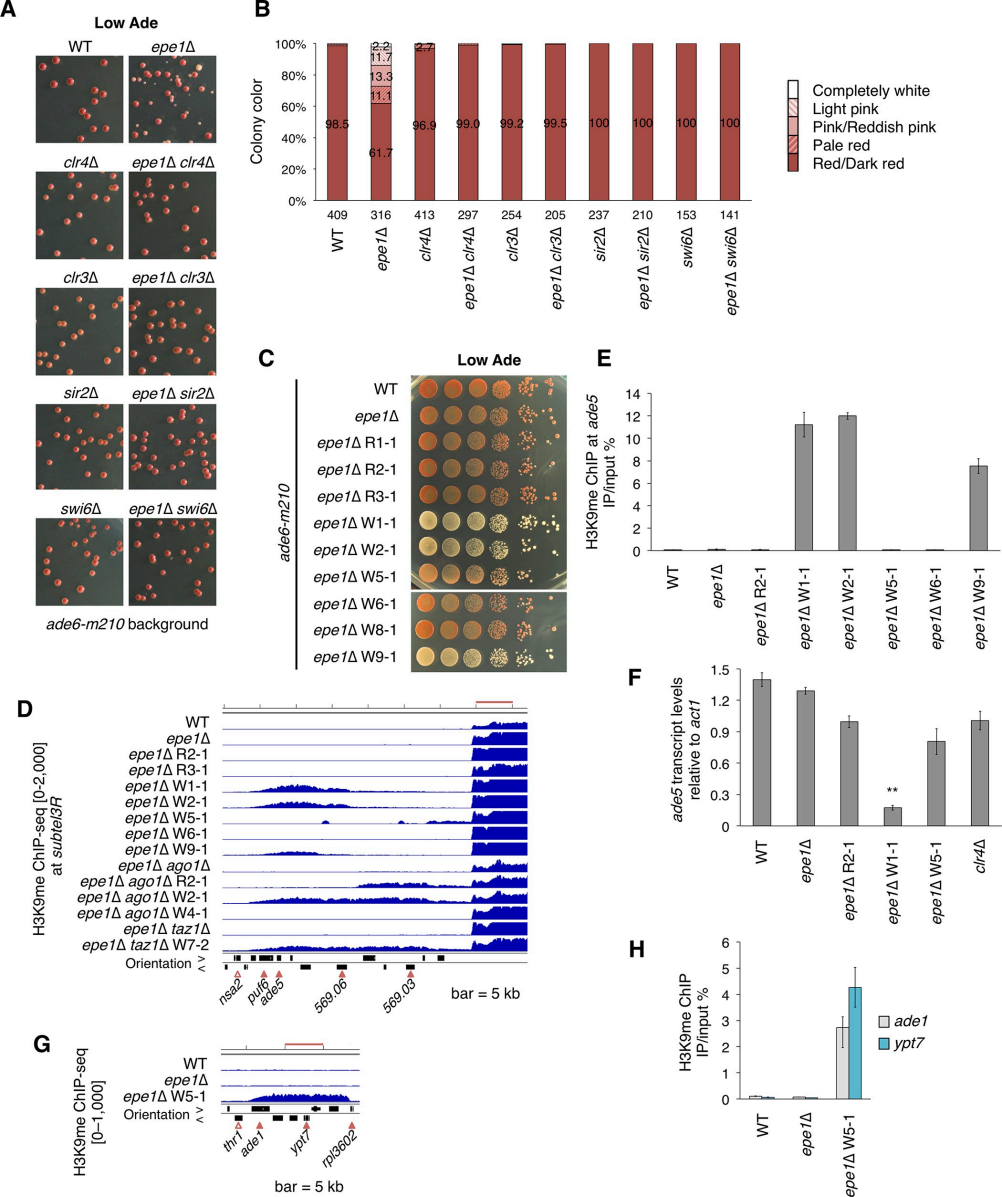
The red-white variegation phenotype is linked to ectopic heterochromatin formation. (A) Comparison of colony color among double deletion mutants on adenine-limited medium. These strains had no *ade6*^+^ marker. Endogenous *ade6* is disrupted by a loss-of-function mutation (*ade6-m210*) [29]. Strains with single deletions of major heterochromatin assembly factors are shown side by side. Eight percent of the plate area is displayed. (B) Percentage of colored and white colonies of single and double deletion mutants shown in (A). (C) Ten-fold serial dilution assay. Some of the obtained isolates were spotted on adenine-limited medium. *epe1*Δ W6-1 and W8-1 clones displayed parental *epe1*Δ-like phenotypes despite white colony isolation. (D) ChIP-sequencing (ChIP-seq) analysis of H3K9me. Right subtelomeres of chromosome III are shown. Blue graphs indicate normalized fragment counts. The vertical range of the graphs is indicated on the left. Open arrowhead, essential genes based on the *S*. *pombe* gene database (PomBase; https://www.pombase.org); filled arrowhead, nonessential genes; orientation >, left to right; orientation <, right to left. Bar, 5 kb. (E) ChIP-qPCR analysis of H3K9me at *ade5*. (F) qRT-PCR analysis of *ade5* transcript levels. **p < 0.05. *p*-values were determined using a two-tailed Student’s *t*-test comparing the indicated sample value with the value of other samples. (G) ChIP-sequencing analysis of H3K9me. *ade1* and the surrounding region are shown. Bar, 5 kb; open arrowhead, essential genes based on PomBase; filled arrowhead, nonessential genes. (H) ChIP-qPCR analysis of H3K9me at *ade1* and *ypt7*. ChIP-qPCR and qRT-PCR data are represented as mean ± SD of three independent experiments (n = 3).

The features of W70 mentioned above prompted us to speculate that loss of Epe1 induces the white phenotype independently of *otr1R*::*ade6*^+^ via ectopic heterochromatin formation at the *ade5* locus, although a large part of the red-white variegation in *epe1Δ* cells depended on variegated expression of *otr1R*::*ade6*^+^. To test this conjecture, we used a strain harboring *ade6-m210*, which has no functional allele of the *ade6* gene. The strain showed a uniform red phenotype on adenine-limited medium. Introduction of *epe1*Δ induced a red-white variegated phenotype with a lower proportion of pink and white colonies than that of the *epe1*Δ *otr1R*::*ade6*^+^ strain (Fig 2A and 2B).

We hypothesized that ectopic heterochromatin formation was responsible for the white phenotype in the *epe1*Δ *ade6-m210* strain and that its stochastic formation resulted in red-white variegation. To investigate this hypothesis, we introduced mutations of genes required for heterochromatin formation and analyzed their effects on the variegation phenotype (Fig 2A and 2B). Loss of Clr4 abolished red-white variegation in the *epe1*Δ *ade6-m210* background, indicating a requirement for the H3K9 methyltransferase Clr4. *clr3* and *sir2*, which encode histone deacetylases, are required for self-propagation of heterochromatin [30–34]. The introduction of *clr3*Δ or *sir2*Δ into the *epe1*Δ background also induced a uniform red phenotype. Similarly, loss of Swi6 suppressed variegation. These results suggest that red-white variegation relies on heterochromatin assembly.

We next examined the requirement for Ago1 and Taz1 for *epe1*Δ-induced variegation, because both factors are involved in subtelomeric constitutive heterochromatin formation [5]. *epe1*Δ *ago1*Δ, *epe1*Δ *taz1*Δ, and *epe1*Δ *ago1*Δ *taz1*Δ strains displayed red-white variegated phenotypes (S2A and S2B Fig), indicating that neither RNAi nor Taz1 was essential for *epe1*Δ-induced variegation.

To confirm the relationship between the red-white variegation and stochastic ectopic heterochromatin formation, we established red- and white-isolated clones from *ade6-m210* strains harboring *epe1*Δ, *epe1*Δ *ago1*Δ, and *epe1*Δ *taz1*Δ (Fig 2C). Using ChIP-sequencing, we analyzed the whole genome distribution of H3K9me of these strains. The results revealed formation of strong ectopic heterochromatin on *ade5* in *epe1*Δ W1-1, W2-1, and W9-1, which was associated with decreased *ade5* expression (Fig 2D–2F). This suggests that *ade5* ectopic heterochromatin caused the appearance of white colonies of *epe1*Δ *ade6-m210* cells. *epe1*Δ *ago1*Δ W2-1 and *epe1*Δ *taz1****Δ*** W7-2 also harbored *ade5* ectopic heterochromatin, indicating that neither RNAi nor Taz1 was required for ectopic heterochromatin formation at the *ade5* region (Fig 2D, S2C Fig).

The *epe1*Δ W5-1 strain displayed a pink phenotype without increased H3K9me on *ade5*, but displayed ectopic heterochromatin formation on *ade1* and its neighboring region, and a decreased level of *ade1* expression (Fig 2G and 2H, S2D Fig). Since, like Ade5, Ade1 functions upstream of Ade6 in *de novo* IMP biosynthesis (Fig 1B), the pink phenotype was probably due to ectopic silencing of *ade1*. Accordingly, red isolates showed no stable pink/white phenotype or ectopic heterochromatin formation on pigmentation-associated genes. These results suggest that white colonies are generated when ectopic heterochromatin is formed at pigmentation-associated genes.

### Ectopic heterochromatin formation induces a variegated phenotype of carbon source utilization

**Fig 3:**
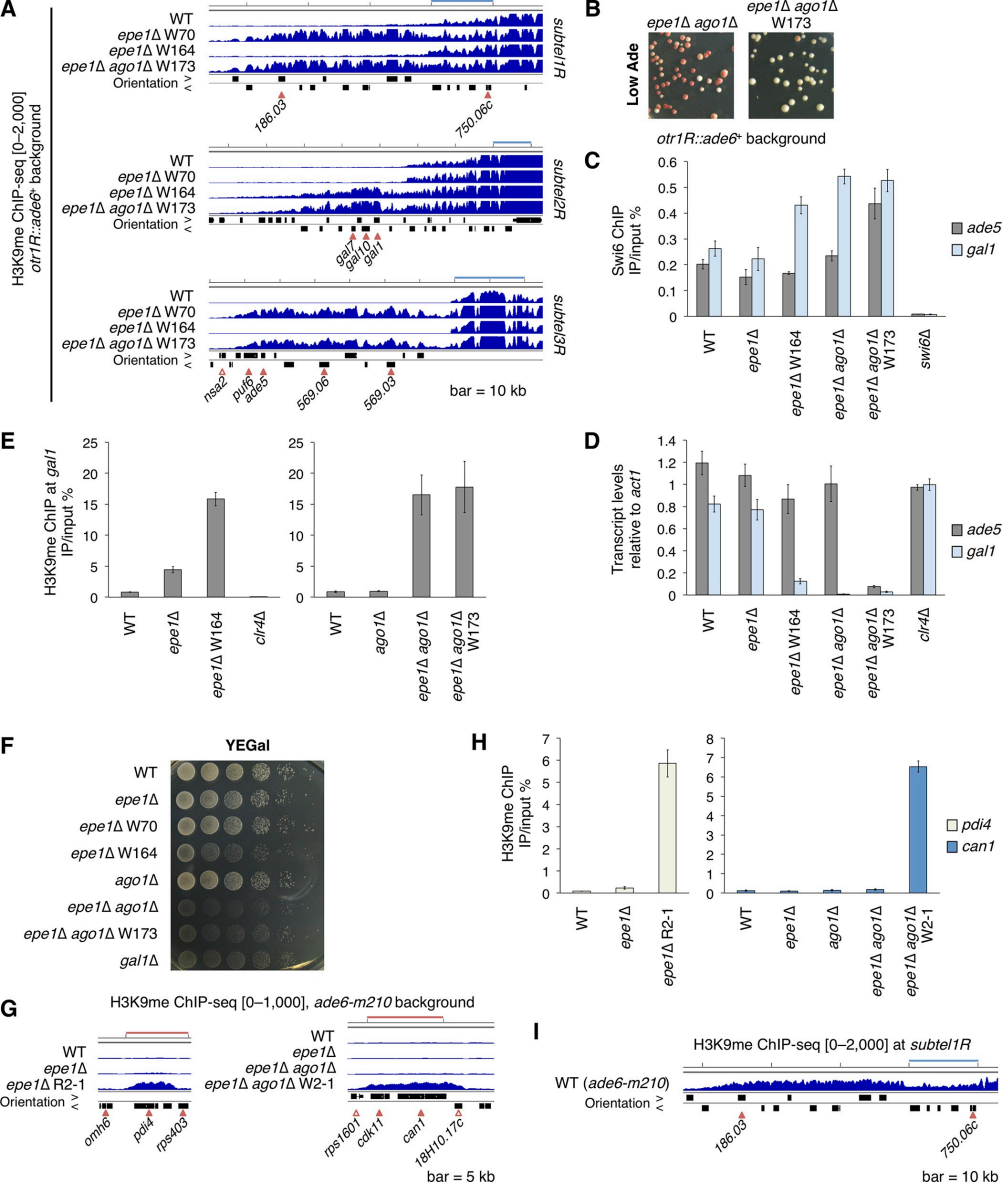
Stochastic formation of ectopic heterochromatin and development of islands constitute the diversified epigenotypes of the *epe1*Δ strain. (A) ChIP-sequencing analysis of H3K9me in *otr1R*::*ade6*^+^ strains. Three right subtelomeric regions are shown. The vertical range of the graphs is indicated on the left. Bar, 10 kb; open arrowhead, essential genes based on the *S*. *pombe* gene database; filled arrowhead, nonessential genes; orientation >, left to right; orientation <, right to left. (B) Colony color of *epe1*Δ *ago1*Δ and *epe1*Δ *ago1*Δ W173 strains on adenine-limited medium. (C) ChIP-qPCR analysis of Swi6 at *ade5* and *gal1* in *epe1*Δ W164 and *epe1*Δ *ago1*Δ W173 clones. (D) qRT-PCR analysis of *ade5* and *gal1* transcript levels. (E) ChIP-qPCR analyses of H3K9me at *gal1* in *epe1*Δ W164 and *epe1*Δ *ago1*Δ W173 clones. (F) Ten-fold serial dilution assay on YEGal medium. (G) ChIP-sequencing analysis of H3K9me. *pdi4* and *can1* and their surrounding regions are shown. Bar, 5 kb. (H) ChIP-qPCR analyses of H3K9me at *pdi4* and *can1*. (I) ChIP-sequencing analysis of H3K9me in the *ade6-m210* WT strain. *subtel1R* is displayed. Bar, 10 kb. ChIP-qPCR and qRT-PCR data are represented as mean ± SD of three independent experiments (n = 3). The separated data in (E) and (H) were obtained from two independent analyses.

We also performed H3K9me ChIP-seq analysis in previously isolated W70 and W164 strains (*otr1R*::*ade6*^+^ background). Despite having the same parental strain, *epe1*Δ W70 and W164 clones did not share ectopic heterochromatin at right subtelomeres (Fig 3A). W70 had large ectopic heterochromatin at *subtel1R* and *3R*, while W164 had it at *subtel2R*. The result suggested that these ectopic heterochromatin domains had been differentially established in the *epe1Δ* cell mixture. We additionally obtained a W70-like white-isolated clone from the *otr1R*::*ade6*^+^ *epe1*Δ *ago1*Δ strain, designated as W173 (Fig 3B, S3A Fig). In contrast to *epe1*Δ W70 and W164, *epe1*Δ *ago1*Δ W173 had ectopic heterochromatin at *subtel1R*, *2R*, and *3R* (Fig 3A, 3C and 3D, S3B Fig). We noticed that the *gal1* gene was embedded in *subtel2R* ectopic heterochromatin formed in *epe1*Δ W164 and *epe1*Δ *ago1*Δ W173 clones (Fig 3A and 3E). Swi6 levels on *gal1* were higher and *gal1* transcript levels were substantially lower in these mutants than in the WT (Fig 3C and 3D), and cells harboring *gal1* ectopic heterochromatin displayed defective growth on galactose-containing medium (Fig 3F), indicating that *gal1* was silenced by ectopic heterochromatin. This implies that ectopic heterochromatin can affect phenotypes other than red pigment formation. Interestingly, the parental strain of *epe1*Δ *ago1*Δ W173 had *gal1* ectopic heterochromatin (Fig 3E), indicating that *subtel2R* ectopic heterochromatin already existed in the parental strain and that ectopic heterochromatin at *subtel2R* and *3R* had been established at different times.

### Development of heterochromatin islands and formation of ectopic heterochromatin constitute the diversified epigenotypes of the *epe1*Δ strain

Loss of Epe1 increases H3K9me levels at heterochromatin islands [17, 23, 24, 35, 36]. We observed heterogeneous development of islands among clones harboring the *ade6-m210* background (S3C Fig). Note that some of the islands were hardly detected in the WT strains and a large amount of H3K9me stochastically accumulated among isolated *epe1*Δ clones (for example, *Is 3*, *8*, *19* in S3C Fig). These results suggest that some islands represent an H3K9me source that is inconsistently active.

The red-isolated *epe1*Δ R2-1 and white-isolated *epe1*Δ *ago1*Δ W2-1 clones harbored ectopic heterochromatin on pigmentation-unrelated genes, *pdi4* and *can1*, respectively, both of which did not correspond to known euchromatic H3K9me peaks [23, 24, 37–42], and had no H3K9me in their original WT cells (Fig 3G and 3H, S3D Fig). This suggests that unknown potential H3K9me sources still existed in the *S*. *pombe* genome and strong ectopic heterochromatin can be established at these source-positive sites. In addition, *epe1*Δ *taz1*Δ W7-2 cells harbored ectopic heterochromatin at *lys1* (*cen1L*) and *clr2* loci (S3C Fig). Other ectopic H3K9me peaks in clones are shown in S2 and S3 Tables. These results showed that each *epe1*-null cell had a distinct epigenotype.

It is noteworthy that the *subtel1R* heterochromatin landscape was different between the WT (*epe1*^*+*^) strains (Fig 3A and 3I): the WT *ade6-m210* strain harbored extended subtelomeric heterochromatin, which was not detected in the WT *otr1R*::*ade6*^+^ strain. The result indicates that the epigenetic profile varies between the WT strains even though they have functional Epe1 and similar genetic backgrounds.

### Epe1 prevents ectopic heterochromatin-mediated red-white variegation via a JmjC domain-independent mechanism

**Fig 4:**
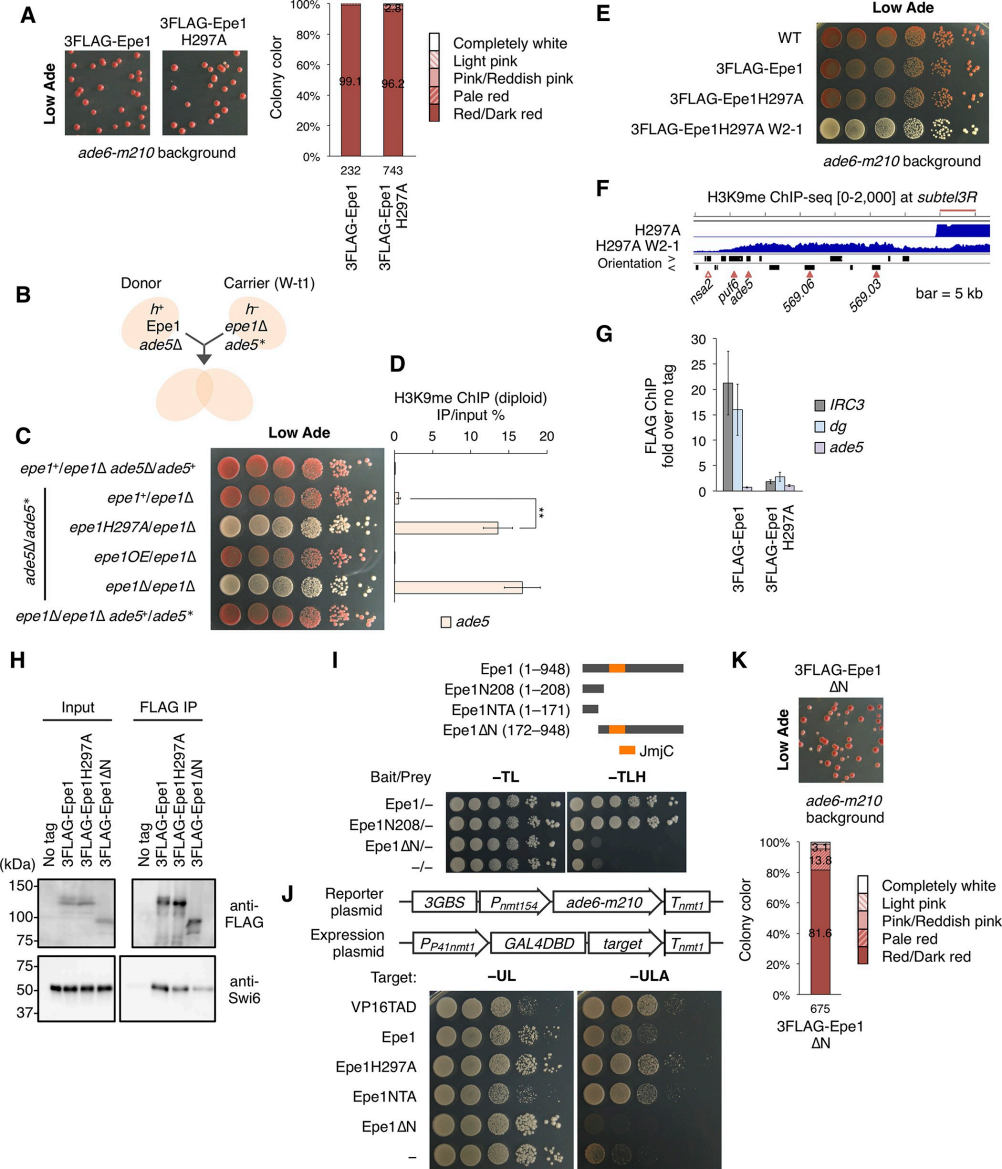
Epe1 prevents the ectopic heterochromatin-mediated red-white variegation via an N-terminal transcriptional activation domain-dependent mechanism. (A) Comparison of colony color of strains expressing the 3FLAG-tagged Epe1 protein and H297A JmjC domain mutant on adenine-limited medium (left). Eight percent of the plate area is displayed. Percentage of colored and white colonies is shown to the right. (B) Schematic representation of the diploid complementation analysis. The *epe1*Δ W-t1 strain was used as a carrier strain, which harbored *ade5* ectopic heterochromatin (ade5*). (C) Ten-fold serial dilution assay for diploid strains. The indicated strains were spotted onto adenine-limited medium. (D) ChIP-qPCR analysis of H3K9me for diploid cells at *ade5*. The qPCR signals were monoallelic. **p < 0.05 (two-tailed Student’s *t*-test). (E) Ten-fold serial dilution assay of 3FLAG-Epe1H297A and W2-1 white-isolated strains. (F) ChIP-sequencing analysis of H3K9me. *subtel3R* is shown. Bar, 5 kb. (G) ChIP-qPCR analysis of FLAG-tagged Epe1 proteins at *IRC3*, *dg*, and *ade5*. *IRC3*, the boundary sequence located outside *cen3*. WT cells were used for the no tag control. (H) Co-immunoprecipitation of Swi6 with Epe1 or its mutants. Immunoprecipitated Epe1 and Swi6 were detected by Western blotting. Input represents 20% (FLAG) or 0.4% (Swi6) of the amount of lysates used for immunoprecipitation. (I) Analysis of transcriptional activation activity of Epe1 using the *HIS3* reporter gene in the yeast two-hybrid system. Ten-fold serial dilution assay was performed. Epe1 or its truncated mutants were expressed as bait. No protein was expressed as prey. The position of the JmjC domain is indicated in the truncation map. (J) Tethered transcription analysis of the *ade6-m210* reporter in fission yeast. Ten-fold serial dilution assay was performed. Strains containing indicated reporter and expression plasmids were spotted on PMG-based synthetic media. Minus UL, lacking Ura and Leu; −ULA, lacking Ura, Leu, and Ade. The reporter plasmid contained three Gal4-binding sites (*3GBS*) derived from the *GAL1-10* upstream activating sequence. Target peptides indicated were fused to Gal4 DNA binding domain (DBD). The VP16 transactivation domain (TAD) was used as a positive control. NTA, N-terminal transcriptional activation domain (1–171 amino acids region). (K) Colony color of a strain expressing the 3FLAG-tagged Epe1ΔN protein (left). This strain harbored the *ade6-m210* background. Percentage of colored and white colonies is shown (right). ChIP-qPCR data are represented as mean ± SD of three independent experiments (n = 3).

Since Epe1 promotes demethylation of artificially deposited H3K9me *in vivo* in a JmjC domain-dependent mechanism [25, 26], we asked whether Epe1 demethylation plays a role in suppression of the red-white variegation observed in Fig 2A. To examine this, we generated strains expressing N-terminal tagged wild-type Epe1 (3FLAG-Epe1) and Epe1H297A, which harbors an alanine substitution at the first Fe^2+^-binding residue [10, 13]. H297A is a canonical catalytic-dead mutant that lacks *in vivo* demethylation activity on artificially deposited H3K9me [25, 26]. Both proteins were expressed from the endogenous *epe1* promoter and displayed almost the same protein expression levels (S4A Fig). Unlike loss of Epe1, the H297A mutation generated few pink/white colonies in the *ade6-m210* background (Fig 4A); indeed, 96.2% of Epe1H297A cells formed WT-like red colonies, while 61.7% of *epe1*Δ cells did. This result indicates that Epe1 almost fully suppressed ectopic heterochromatin-mediated variegation in a JmjC domain-independent manner.

We have already shown that introduction of *epe1*^+^ into W70 cells led to a red phenotype via the removal of H3K9me at ade5* (Fig 1H and 1I); however, Epe1 suppressed variegation independently of JmjC activity. Thus, we next analyzed whether the JmjC domain is required for the removal of H3K9me at ade5*, using a diploid complementation assay (Fig 4B–4D). We constructed a carrier strain (*epe1*Δ W-t1) bearing *ade5* ectopic heterochromatin (ade5*) transferred from the *epe1*Δ *ago1*Δ W173 strain, and confirmed the retention of *subtel3R* ectopic heterochromatin including ade5* after sexual reproduction (S4B and S4C Fig). Introduction of *epe1H297A* as well as *epe1*Δ into *epe1*Δ W-t1 resulted in diploid cells that retained the white phenotype and H3K9me at ade5*, while introduction of *epe1*^+^ complemented the white phenotype and almost depleted H3K9me of ade5* (Fig 4C and 4D). Furthermore, since the anti-H3K9me antibody recognizes mono-, di-, and tri-methylated H3K9, we tested whether *ade5* ectopic heterochromatin contained di- and tri-methylation marks. We detected ectopic H3K9me2 and me3 signals at *ade5* and found that their levels were substantially reduced in Epe1-introduced diploid cells (S4D Fig). These results indicate that Epe1 promoted demethylation of methyl-H3K9 including H3K9me2 and me3 at ectopic heterochromatin in a JmjC-dependent manner. However, despite of the loss of its demethylation function, Epe1H297A suppressed ectopic heterochromatin-mediated variegation (Fig 4A). Therefore, Epe1 has a JmjC domain-independent function that suppresses ectopic heterochromatin formation before accumulation of large amounts of H3K9me.

Epe1H297A cells generated a few pink colonies that were not generated by wild-type cells (Fig 4A). Thus, we suspected that the JmjC domain-independent function cannot completely suppress ectopic heterochromatin formation and that JmjC-dependent demethylation contributes to full suppression to some extent. We isolated a white clone (Epe1H297A W2-1) by replating minor pink colonies generated from Epe1H297A cells (Fig 4E). ChIP-seq analysis revealed that Epe1H297A W2-1 cells had ectopic heterochromatin at the *subtel3R* region (Fig 4F). This suggested that ectopic heterochromatin occurred at a low frequency by escaping JmjC-independent prevention and that JmjC-dependent demethylation removed this ectopic heterochromatin.

In the course of the analysis of Epe1H297A, we examined whether the H297A mutation affected Epe1 localization at heterochromatin. FLAG ChIP analysis revealed that H297A reduced appreciably Epe1 enrichment on centromeric *dg* repeats and *IRC3* (Fig 4G), a centromeric boundary sequence where Epe1 accumulates to a high level [12, 14]. By contrast, H3K9me was maintained at *IRC3* and *dg* (S4E Fig). Although the Swi6 level was significantly reduced, it was still present at both regions (S4F Fig), suggesting that the reduced Swi6 level may not have been the primary cause of the drastic reduction in Epe1H297A enrichment on heterochromatin. We next validated the physical interaction between the Epe1H297A mutant and Swi6 by yeast two-hybrid analysis. Because budding yeast has no endogenous H3K9me or HP1, neither of these factors could affect the analysis. The H297A substitution slightly impaired the interaction (S4G Fig), which was confirmed by the results of a bait-prey exchange experiment. We performed co-immunoprecipitation analysis of Swi6 with Epe1H297A. Consistent with the results of yeast two-hybrid assay, the Epe1H297A mutant interacted with Swi6 with a slightly lower efficiency than wild-type Epe1 (Fig 4H). Together, these results showed that mutation of the Fe^2+^-binding residue in the JmjC domain slightly impaired the physical interaction between Epe1 and Swi6, but largely reduced targeting of Epe1 to heterochromatin. In addition, these results showed that heterochromatin association activity of Epe1 was not important for the JmjC-independent prevention of ectopic heterochromatin formation.

However, how Epe1 finds target sites to prevent ectopic heterochromatin formation is unknown. Since Epe1 physically interacts with the bromodomain protein Bdf2, which is required for heterochromatin-euchromatin boundary formation [14], we predicted that Bdf2 would recruit Epe1 to the target sites. However, *bdf2*Δ cells showed an almost uniform red phenotype in the *ade6-m210* background (S4H Fig), suggesting that Bdf2 was not related to suppression of variegation and ectopic heterochromatin formation.

### N-terminal transcriptional activation domain is involved in the prevention of ectopic heterochromatin formation

In the yeast two-hybrid system, the Epe1 protein expressed as bait activates transcription of reporter genes without prey [15] (Fig 4I). We found that deletion of the N-terminal 171 amino acids (Epe1ΔN) abolished transcriptional activation by Epe1 and the N-terminal 208 amino acids (Epe1N208) activated transcription of the *HIS3* reporter independently of JmjC (Fig 4I), suggesting that the N-terminal 171 amino acids region is required for the transcriptional activation activity.

To examine whether the N-terminal 1–171 amino acids region has a transcriptional activation function in fission yeast, we established a plasmid-based reporter system (Fig 4J). We constructed a reporter plasmid containing the coding sequence of *ade6-m210* transcribed from 154 bases of the *nmt1* promoter combined with three copies of Gal4 binding sites instead of its thiamine regulatory element [43]. We also constructed expression plasmids for expressing Epe1 or its mutants fused to the Gal4 DNA binding domain (Gal4DBD). These plasmids were introduced into a fission yeast strain harboring the *ade6-M216* allele. Since *ade6-m210* and *ade6-M216* alleles show intragenic complementation, cells expressing *ade6-m210* would grow on medium without adenine, like *ade6*^+^ cells. Indeed, cells expressing the VP16 transactivation domain (TAD), a well-characterized transcriptional activation domain, fused to Gal4DBD grew on medium without adenine, while cells expressing Gal4DBD alone did not (Fig 4J). Cells expressing Epe1, Epe1H297A, and NTA fused to Gal4DBD, but not Epe1ΔN, showed Ade^+^ phenotypes (Fig 4J). This indicated that the NTA domain (1–171 amino acids region) harbored transcriptional activation activity in fission yeast. Note that expression of Gal4DBD-VP16TAD or -NTA fusion protein induced growth defect on the control plate, which is likely caused by off-target effects of Gal4DBD fusion proteins with a strong activation domain.

We introduced Epe1ΔN into *ade6-m210* cells to examine the effect of the ΔN mutation on the suppression of ectopic heterochromatin formation. Epe1ΔN cells formed pink/white colonies with a slightly lower frequency than *epe1Δ* cells (Fig 4K), indicating that the NTA domain contributed to the suppression of ectopic heterochromatin-mediated variegation. We performed co-immunoprecipitation analysis of Swi6 with Epe1ΔN and found that Epe1ΔN interacted with Swi6 with much lower efficiency than wild-type Epe1 (Fig 4H). However, consistent with the previous report [13], the C-terminal half of Epe1 (487–948 amino acids region) interacted with Swi6 in the yeast two-hybrid system, but the N-terminal half (1–486) did not (S4I Fig). These results suggest that the C-terminal half contains a Swi6 binding site and the NTA domain indirectly contributes to Epe1–Swi6 interaction. In summary, our results suggest that the NTA domain in Epe1, rather than its JmjC domain or heterochromatin association, is required for the prevention of ectopic H3K9me deposition. By contrast, the intact JmjC domain of Epe1 is required for the removal of already-established ectopic heterochromatin.

### JmjC-mediated incomplete suppression of ectopic heterochromatin provides metastable epigenetic variation

**Fig 5:**
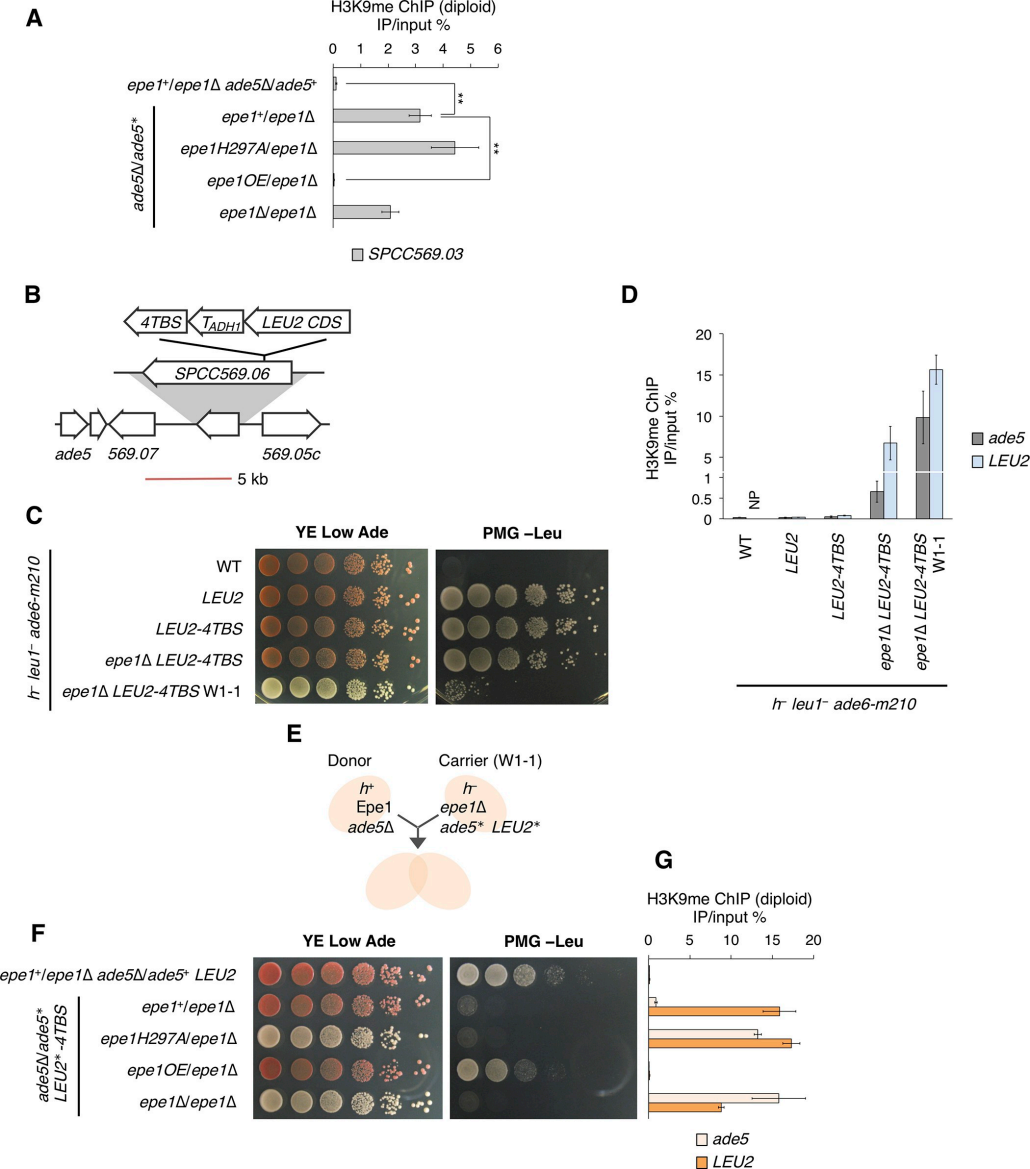
JmjC-mediated incomplete suppression of ectopic heterochromatin provides metastable epigenetic variation. (A) ChIP-qPCR analysis of H3K9me at *SPCC569*.*03* in diploid cells. The indicated strains provided biallelic signals. **p < 0.05 (two-tailed Student’s *t*-test). The strains used were the same as those in Fig 4C and 4D. (B) Schematic diagram of the *LEU2-4TBS* invasion system. Transcription of the coding sequence of *S*. *cerevisiae LEU2* was initiated by the promoter of *SPCC569*.*06* and terminated by the *S*. *cerevisiae ADH1* terminator. *4TBS* was placed after the terminator. Bar, 5 kb. (C) Ten-fold serial dilution assay of the strain harboring *4TBS*-induced heterochromatin shown on adenine-limited and PMG −Leu media. (D) ChIP-qPCR analysis of H3K9me at *ade5* and *LEU2*. NP, not performed. *4TBS* is about 1.3 and 9.5 kb away from the PCR loci of *LEU2* and *ade5*, respectively. (E) Schematic diagram of diploid complementation of ade5* and LEU2* epialleles. (F) Ten-fold serial dilution assay on adenine-limited and PMG −Leu media for diploid complementation. (G) ChIP-qPCR analysis of H3K9me for diploid strains at *ade5* and *LEU2*. All strains provided monoallelic signals. ChIP-qPCR data are represented as mean ± SD of three independent experiments (n = 3).

As shown in Fig 4D, introduction of a single copy of *epe1*^+^ almost completely removed H3K9me on ade5*. However, H3K9me as well as specific H3K9me2 and H3K9me3 marks on a *subtel3R* gene, *SPCC569*.*03*, were maintained after introduction of *epe1*^+^ (Fig 5A, S4D Fig). Importantly, no H3K9me accumulation was observed on *SPCC569*.*03* before deletion of *epe1* (S1E Fig), indicating that ectopic heterochromatin formation at *subtel3R* was a partially irreversible epigenetic alteration and that Epe1 selects the position for demethylation.

We next asked whether an increase in the level of Epe1 would reduce persistent ectopic H3K9me. Introduction of an Epe1 overexpression (Epe1OE) allele achieved complete removal of H3K9me on *SPCC569*.*03*, suggesting that the amount of Epe1 is critical for the removal of residual H3K9me (Fig 5A).

After re-introduction of Epe1 into *epe1*Δ ade5* cells, we observed a difference in persistent H3K9me levels between *ade5* and *SPCC569*.*03*, both of which had not undergone H3K9me deposition in the presence of Epe1 (Figs 4D and 5A). Since *SPCC569*.*03* is close to subtelomeric constitutive heterochromatin, we speculated that Epe1 would not remove already-established heterochromatin neighboring a constitutive supply source of H3K9me. To investigate this, we used a mild H3K9me source that does not deposit H3K9me in the presence of Epe1 but does in the absence of Epe1. Taz1, a subunit of the telomere protection complex Shelterin, binds to telomeric repeats [44, 45]. Five heterochromatin islands harbor 2–5 copies of telomere repeat units coupled with a late replication origin, which recruit Clr4 via Shelterin to deposit H3K9me [36, 46]. Thus, we predicted that low numbers of telomere repeats lacking the late origin would induce little or no H3K9me accumulation when Epe1 was present, but initiate H3K9me deposition when Epe1 was absent. We constructed a Taz1-binding cassette (*LEU2*-*4TBS*), which consists of the coding sequence (CDS) of *S*. *cerevisiae LEU2* and four copies of a unit of telomere repeats [44, 47], and introduced it into the *ade6-m210* background strain, where it was inserted after the *SPCC569*.*06* promoter, a location distant from *ade5* (Fig 5B). *S*. *cerevisiae LEU2* functions in place of *S*. *pombe leu1*. The inserted *LEU2* complemented the *leu1*^−^ allele harbored by the *ade6-m210* strains, resulting in growth on −Leu medium (Fig 5C). The *epe1*Δ *LEU2-4TBS* strain displayed marginal growth retardation on −Leu medium, indicating that *4TBS* did not strongly silence *LEU2* even in the absence of Epe1. This strain also showed red-white variegation. White colony isolation constantly generated clones that displayed Leu^−^ and irreversible white phenotypes. One of the clones, *epe1*Δ *LEU2*-*4TBS* W1-1, harbored higher H3K9me deposition on *ade5*, *LEU2*, and surrounding genes (Fig 5C and 5D, S5A Fig). We designated the silenced *ade5* and *LEU2* alleles as ade5* and LEU2*, respectively. Diploid-based complementation showed that introduction of wild-type Epe1 resulted in a red colony and Leu^−^ phenotype (Fig 5E and 5F). Consistently, H3K9me on *ade5* was almost completely removed, whereas that on *LEU2* and *SPCC569*.*03* was not reduced by the provision of a single copy of Epe1 (Fig 5G, S5B Fig), suggesting that Epe1 failed to antagonize heterochromatin neighboring the H3K9me source but greatly decreased H3K9me levels at non-contiguous locations. By contrast, introduction of Epe1OE resulted in a red colony and Leu^+^ phenotype and the complete removal of *subtel3R* ectopic H3K9me, indicating that Epe1OE overcame *4TBS*-induced self-retaining heterochromatin. Constitutive heterochromatin harboring a strong maintenance system such as RNAi, in contrast to weak systems such as *4TBS*, withstands Epe1 overexpression to some extent [12, 17]. Our results suggest that the balance between the level of Epe1 and intensity of the self-maintenance system in each domain determines the extent of heterochromatin retention. Importantly, *LEU2*-*4TBS* deposited no H3K9me on *LEU2* or *ade5* in the *epe1*^+^ background, whereas accumulation was obvious in *epe1*Δ (Fig 5D). This strongly suggests the existence of a mechanism for the retention of altered chromatin structure, in which Epe1 suppresses *de novo* ectopic heterochromatin establishment, but after establishment via escape from suppression, the ectopic heterochromatin is retained by the latent H3K9me source despite the presence of Epe1. In summary, we reveal that Epe1 allows retention of robust ectopic heterochromatin, in which the level of persistent ectopic H3K9me is determined by the amount of Epe1, the distance between target H3K9me and the H3K9me source, and the intensity of the source.

## Discussion

**Fig 6:**
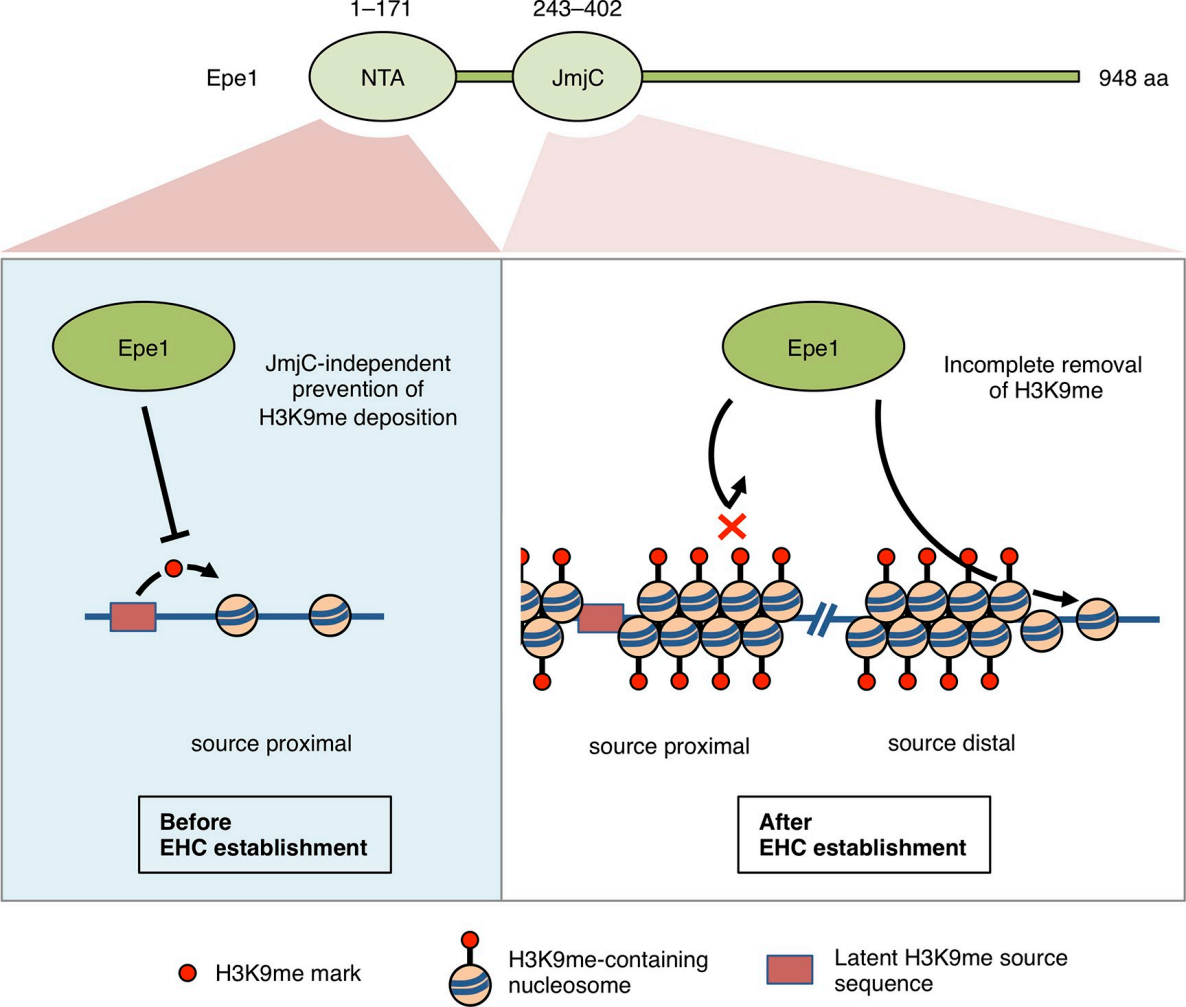
Model of two distinct functions of Epe1 in the suppression of ectopic heterochromatin formation and selective retention of robust ectopic heterochromatin. Before ectopic heterochromatin establishment (left), Epe1 prevents early deposition of H3K9me via a mechanism that involves its N-terminal transcriptional activation (NTA) domain but not its JmjC. After ectopic heterochromatin establishment (right), Epe1 promotes incomplete demethylation of ectopic H3K9me. Epe1 cannot disrupt already-established ectopic heterochromatin near an H3K9me supply source because the source provides H3K9me and counterbalances removal of H3K9me by Epe1, whereas Epe1 can remove the source-distal H3K9me mark in a JmjC-dependent manner. EHC, ectopic heterochromatin. NTA domain, 1–171 amino acid region as identified in this study; JmjC domain, 243–402 amino acid region as assigned in the SMART database (http://smart.embl-heidelberg.de).

In this study, we demonstrated that Epe1 suppressed ectopic heterochromatin formation via two mechanisms as shown in Fig 6. First, Epe1 prevents the initial deposition of H3K9me at potential ectopic heterochromatin formation sites via a mechanism that does not require the function of its JmjC domain. Second, Epe1 promotes demethylation of H3K9me from established ectopic heterochromatin via a mechanism that requires its JmjC domain. These two distinct mechanisms cooperate to suppress ectopic heterochromatin formation. Loss of Epe1 induced stochastic accumulation of H3K9me, resulting in heterogeneous ectopic heterochromatin formation among clonal cells. Because the demethylation function of Epe1 is antagonized by a constitutive supply of H3K9me, some of the ectopic heterochromatin established by escape from prevention can be retained and provide a basis for epigenetic differences.

### JmjC- and heterochromatin association-independent prevention

The JmjC mutant Epe1H297A almost completely suppressed the red-white variegation induced by ectopic heterochromatin assembly, while it failed to remove already-established ectopic heterochromatin (Fig 4A and 4D). Therefore, Epe1 prevents H3K9me deposition before the establishment of ectopic heterochromatin. Although *epe1H297A* cells largely maintained heterochromatin at *IRC3* and *dg* (S4E and S4F Fig), Epe1H297A largely lost its heterochromatin localization (Fig 4G). Nevertheless, Epe1H297A prevented ectopic heterochromatin formation. This suggests that Epe1 recognizes potential ectopic heterochromatin formation sites in a manner distinct from heterochromatin targeting. However, the targeting mechanism is unclear at this stage.

Although no domain other than JmjC had been found in Epe1, the newly identified NTA domain showed transcriptional activation ability in both budding and fission yeasts (Fig 4I and 4J). These results show that the NTA domain functions as a conserved transcriptional activation domain like VP16 TAD to recruit RNA polymerase II. Accordingly, Epe1 interacts with a histone acetyltransferase complex, SAGA, which is involved in transcriptional activation [48]. Importantly, loss of the NTA domain induced a variegation phenotype (Fig 4K). These results raise the possibility that the transcriptional activation activity of Epe1 is involved in the prevention of H3K9me deposition. Recent reports show that loss of Leo1, a component of the transcription elongation complex Paf1C, causes ectopic heterochromatin formation [37, 49, 50]. Similarly, loss of Mst2, a histone acetyltransferase, induces ectopic heterochromatin formation [24]. Importantly, loss of each of these proteins causes a decrease in histone turnover [24, 50]. Since histone turnover is associated with transcription [50, 51] and Epe1 promotes histone turnover in heterochromatin [18], Epe1 might exclude H3K9me-containing nucleosomes by activating histone turnover coupled with transcriptional activation at the potential ectopic heterochromatin formation sites. It is still possible that the NTA domain has a function, other than transcriptional activation, which contributes to the prevention of ectopic heterochromatin formation. We found that lack of the NTA domain impaired the interaction of Epe1 with Swi6 (Fig 4H). Heterochromatin association is not required for the prevention of ectopic H3K9me deposition as described above, but Epe1–Swi6 interaction might contribute to the prevention through a mechanism independent of heterochromatin association. Further studies such as determination of important amino acids for the transcriptional activation and interaction with Swi6 will clarify the mechanism of JmjC-independent prevention.

### Accurate metal binding by the JmjC domain is required for heterochromatin association

The conserved histidine at residue 297 in the JmjC domain, which is important for Fe^2+^ binding, is essential for the promotion of H3K9 demethylation by Epe1 *in vivo* (Fig 4D) [25, 26]. We found that histidine 297 was required for localization to heterochromatin (Fig 4G). We also found that the H297A mutation slightly impaired the interaction of Epe1 with Swi6, although this may not fully explain the reduced heterochromatin localization of Epe1 (Fig 4H, S4G Fig). In contrast, Swi6 interacted with the C-terminal half of Epe1, which lacks the JmjC domain (S4I Fig) [13]. Swi6 is shown to interact with a JmjC mutant, Epe1Y307A, which retains the metal-binding residues [12]. Thus, we speculate that conformational changes in the JmjC domain induced by perturbations in Fe^2+^ binding result in a slight alteration of the interaction surface for Swi6 binding, while severely disrupting the structure of a region essential for heterochromatin association. Heterochromatin association of Epe1 might require another mechanism in addition to its binding to Swi6. Combined with the result of Epe1ΔN–Swi6 interaction analysis (Fig 4H), it is possible that the N-terminal half region of Epe1 contributes to proper conformation for Epe1–Swi6 interaction and heterochromatin association.

### Loss of demethylase enhances stochastic formation of ectopic heterochromatin

Analysis of the H3K9me distribution of isolated *epe1Δ* clones by ChIP-seq revealed that each clone had a unique H3K9me landscape. Thus, ectopic heterochromatin is formed stochastically and maintained stably, but its landscape occasionally shifts to another state. Therefore, we could detect several metastable heterochromatin landscapes among *epe1*Δ isolates.

Ectopic heterochromatin domains formed in *epe1Δ* cells seemed to have preferred positions on the genome. In addition to heterochromatin islands, we found novel ectopic heterochromatin domains such as *clr2*, *pdi4*, *ade1*, and *can1* (Figs 2G and 3G, S3C Fig), which appeared to have no identifiable H3K9me source, except for convergent genes [23, 24, 36, 38–40]. In addition, many ectopic heterochromatin domains identified in several mutant backgrounds have no known H3K9me source [23, 24, 37, 42], while polymerase pausing is an emerging heterochromatin-inducible mechanism [41]. These facts suggest that many potential H3K9me sources for heterochromatin formation exist in the genome and are incidentally activated to form ectopic heterochromatin.

Loss of H3K9 demethylase in other organisms commonly alters traits by perturbing the epigenomic state: in male mice, stochastic formation of testes, ovaries, and testis-ovary hybrids is induced by perturbations in the levels of histone modifications on the testis-determining gene *Sry* [52, 53]; and in *Arabidopsis*, a variety of developmental defects and genome-wide deposition of H3K9me and 5mC are observed [54, 55]. We further assume that mutations in H3K9 demethylases accelerate intratumoral heterogeneity, which interferes with chemotherapy by generating drug-tolerant subpopulations, because tumor evolution involves increases in repressive and decreases in active histone marks [56, 57], and co-dependency between genetic and epigenetic mutations possibly enhances the heterogeneity [58].

### Demethylases control variation of the H3K9me landscape

We found that re-introduction of single copy Epe1 did not erase ectopic heterochromatin when an H3K9me source existed nearby, while Epe1 overexpression completely erased it. On the other hand, increased levels of Epe1 impair constitutive heterochromatin [12, 15, 17]. The expression level of endogenous Epe1 appears to be appropriately regulated to allow ectopic heterochromatin retention while keeping constitutive heterochromatin intact. Since Epe1 is degraded in S phase by the Cul4-Ddb1^Cdt2^ complex [15] and phosphorylation of Swi6 affects localization of Epe1 to heterochromatin [20], we assume that transient inactivation, loss of expression, or delocalization of Epe1 provides an opportunity to change the heterochromatin landscape. Differences in the H3K9me landscape between wild-type strains might reflect transient changes in Epe1 activity (Fig 3A and 3I). Recently, Gallagher et al. reported that low temperature induces the formation of additional heterochromatin islands including *SPCC569*.*03* [59], where we found robust ectopic heterochromatin that tolerated the re-introduction of a single copy of *epe1*^+^ (Fig 5A). This emergence of islands might be caused by impaired Epe1 function at low temperatures. Such regulatory mechanisms appear to be widely applicable to organisms that have demethylases that erase repressive histone methyl marks. Although the genome contains numerous potentially H3K9me-inducible sequences [60–62], not all of them exhibit H3K9me accumulation. Ectopic heterochromatin formation at these sequences, inducible under specific conditions, might result in unprogrammed epigenetic differences.

Variation of the H3K9me landscape could produce adaptive subpopulations, because the variation could switch metabolic pathways or induce changes in growth to those suited for survival in a particular environment [63]. Indeed, ectopic heterochromatin affects ribonucleotide synthesis (*ade5* and *ade1*) and carbon source metabolism (*gal1*). This study provides insights into the mechanisms of epigenetic diversification and maintenance, which underlie cellular homeostasis and heterogeneous evolution.

## Materials and methods

### Strains and media for *S*. *pombe*

The *S*. *pombe* strains used in this study are listed in S5 Table. The media recipes used were previously described [64, 65]. The isolated clones were obtained by two rounds of single-colony isolation, in which “R” or “W” in the strain name means sequential red- or white-colony isolation. The DNA fragments for gene deletion or tagging were constructed using the polymerase chain reaction (PCR)-based method as previously described [66]. For gene deletion, target genes were replaced by the drug-resistant cassettes *kanMX6*, *hphMX6*, and *natMX6* that confer resistance to G418, hygromycin B, and nourseothricin, respectively. The cassettes for expressing 3FLAG-tagged Epe1 and its mutants were constructed on plasmids. The cassettes were then cut out with *Sma*I and introduced into *S*. *pombe* cells. The 3FLAG tag was placed at the N-terminus of Epe1; C-terminal tagging was avoided because of its effect on Epe1 functions [17, 50]. The four copies of a Taz1 binding sequence (*4TBS*) were represented by 5’-GGGTTACAGGGGTTACAGGGGTTACAGGGGTTACAG-3’, composed of four GGTTACAG sequences combined with guanine stretches [44, 47]. For overexpression of 3FLAG-tagged Epe1 (Epe1OE), the *urg1* promoter and *3FLAG* sequence were inserted between the promoter and the CDS of *epe1*. All integrations were confirmed by PCR. The haploid *h*^+^ and *h*^−^strains composing diploid cells harbor *kanMX6* and *natMX6* at the *epe1* locus, respectively. Diploid cell formation was confirmed by dark magenta color on medium containing 5 mg/L Phloxine B (PB; Nacalai Tesque) and resistance to both 100 mg/L G418 sulfate (Wako) and ClonNAT (nourseothricin dihydrogen sulfate; WERNER BioAgents).

### Serial dilution assay

Saturated cells were adjusted to 1 × 10^8^ cells/mL in sterilized water. For preculture of diploid cells, medium containing 100 mg/L G418 sulfate and ClonNAT was used. Diploid cells were saturated without the antibiotics. The suspended cells were serially 10-fold diluted up to 1 × 10^3^ cells/mL. The suspension (6 or 8 μL) was spotted on YE-based complete media or PMG-based synthetic media. To complement genetic mutations, supplements mix (225 mg/L adenine, uracil, histidine, leucine, and lysine, as final concentration) was added (YES and PMGS). Silencing assays were performed on YE media with adenine-dropout supplements mix (Low Ade) and PMG media with leucine-dropout supplements mix (–Leu). The galactose-containing medium (YEGal) was made by replacement of the major carbon source: 30 g/L of galactose, instead of glucose, was added to YES. For haploid cells, plates were incubated at 30°C for 4 days. For diploid cells, plates were incubated at 30°C for 3 days. No assay medium contained antibiotics.

### qRT-PCR analysis

Cells were cultured in 20 mL of YES to 1 (within ±0.1) × 10^7^ cells/mL. The harvested cells were washed with PBS and stored at −80°C. The cell pellet was suspended in AE buffer (50 mM sodium acetate (pH 5.2) and 10 mM EDTA) containing 1% SDS, and then the equivalent volume of acid phenol was added to the suspension. Total RNA was extracted by a freeze-thaw treatment made up of five cycles of rapid freezing in liquid nitrogen followed by incubation in a water bath at 65°C with vortexing. The RNA was subjected to another acid phenol treatment followed by acid phenol/chloroform and chloroform treatments. RNA was recovered by ethanol precipitation and treated with 5 U of recombinant DNase I (Takara Bio) at 37°C for 30 min. DNase I was removed by acid phenol/chloroform treatment. Using Oligo (dT)_15_ primer, 1 μg of total RNA was reverse transcribed into cDNA with PrimeScript Reverse Transcriptase (Takara Bio) at 37°C for 30 min. Quantitative PCR (qPCR) was performed with SYBR Green I dye on a Thermal Cycler Dice Real Time System TP-850 (Takara Bio). The RT− samples (pseudo-experiments without reverse transcriptase) were also subjected to qPCR. The primer sets are listed in S6 Table. The standard curve for each primer set was created from serially 1-to-1000-fold diluted cDNA samples of *clr4*Δ cells. The signals of RT− samples were low and seldom fell within the standard curve, and consequently no RT− sample was adequately analyzed. Relative concentrations of cDNA based on the standard curve were divided by the concentration of *act1* to determine the transcript levels relative to *act1*. The error bars represent the standard deviation of the mean of three independent experiments (n = 3). Each experiment was independently performed from cell culture to qPCR. Note that *ade6-DN/N* harbors a 153 bp of deletion between *Nco*I sites [28]. The primer set for *ade6* specifically detects the centromere-derived transcripts, avoiding amplification of the truncated allele.

### Microarray analysis

The microarray analysis was performed as described previously [67]. Based on the expression ratio, genes with a fold change >1.5 (upregulated) or <1.5 (downregulated) are highlighted in S1D Fig. This experiment was not repeated.

### ChIP-qPCR analysis

Cells grown to 1 (within ±0.1) × 10^7^ cells/mL in 50 mL of YES were fixed with 1% formaldehyde (Nacalai Tesque) for 20 min at 25°C. Diploid cells were precultured in YES containing 100 mg/L G418 sulfate and ClonNAT and then grown to 5 (within ±0.5) × 10^6^ cells/mL in 50 mL of YES without antibiotics followed by the same formaldehyde treatment as for haploid cells. Quenching of the fixative was performed with 150 mM glycine. The cells were harvested by centrifugation and stored at −80°C. Note that the diploid cells were stored for no more than 1 day. The cells were resuspended in Buffer 1 (50 mM HEPES-KOH (pH 7.5), 140 mM NaCl, 1mM EDTA, 1% Triton X-100 (Nacalai Tesque), and 0.1% sodium deoxycholate (Merck Millipore)) containing a protease inhibitor cocktail, and then homogenized with 30–40 cycles of bead beating for 60 s at 4°C to render them refraction-negative under a light microscope. The cell extracts were centrifuged for 60 min at 21,880 × *g* at 4°C. After discarding the supernatant, the pellets were resuspended in Buffer 1 containing a protease inhibitor cocktail and sonicated with resonant metallic bars for 360 s with Bioruptor UCW-310 (Cosmo Bio) set at 310 W (High level) and cooled to around 4°C. The sonicated cell extracts were centrifuged for 15 min at 21,880 × *g* at 4°C, and the resultant supernatant was recovered. Before IP, Dynabeads M-280 Sheep anti-Mouse or anti-Rabbit IgG (Invitrogen) were washed once with Buffer 1 and incubated with 1 μg of anti-H3K9me (mouse monoclonal, m5.1.1, a kind gift from T. Urano, Shimane University), anti-FLAG (mouse monoclonal, M2, Sigma-Aldrich), anti-Swi6 (rabbit polyclonal, made by S.T.), anti-H3K9me2 (mouse monoclonal, 6D11, a kind gift from H. Kimura, Tokyo Institute of Technology), or anti-H3K9me3 (mouse monoclonal, 2F3, a kind gift from H. Kimura, Tokyo Institute of Technology) [68] antibody for 2 h with mild rotation at 4°C followed by washing with Buffer 1. Note that the anti-H3K9me antibody detects mono-, di-, and tri-methylated H3K9 but not unmethylated H3K9. The beads were incubated with the supernatant for 2 h with mild rotation at 4°C. After IP, the beads were washed twice each with Buffer 1, Buffer 1’ (50 mM HEPES-KOH (pH 7.5), 500 mM NaCl, 1 mM EDTA, 1% Triton X-100, and 0.1% sodium deoxycholate), and Buffer 2 (10 mM Tris-HCl (pH 8.0), 250 mM LiCl, 0.5% NP-40 (Roche), and 0.5% sodium deoxycholate) followed by another wash with Buffer 1. The beads were resuspended in Buffer 1 containing RNase A and incubated for 15 min at 37°C and then incubated in 0.25 mg/mL Proteinase K and 0.25% SDS for 2 h at 37°C to obtain IP samples. For input samples, one-fifth volume of the supernatant applied to IP was equally subjected to RNase A and Proteinase K treatments. IP and input samples were incubated for 12–16 h at 65°C for reverse crosslinking. DNA was extracted by neutral phenol/chloroform treatment and recovered by ethanol precipitation. qPCR was performed with SYBR Green I dye on Thermal Cycler Dice Real Time System TP-850 (Takara Bio). The primer sets are listed in S6 Table. The standard curve for each primer set was created from serially diluted input samples of WT cells. Relative concentrations of IP samples based on the standard curve were divided by those of input samples to determine the IP efficiency (IP/input). For FLAG ChIP analysis, IP efficiency of FLAG-tagged cells was divided by that of no tag (WT) cells to determine fold enrichment (fold over no tag). For Swi6 ChIP analysis, incubation at 18°C for 2 h before fixation, which has been usually done to increase signal intensity in previous studies, was not carried out because low temperature induces ectopic heterochromatin formation [59, 69]. This might have increased background levels. The error bars represent the standard deviation of the mean of three independent experiments (n = 3). Each experiment was independently performed from cell culture to qPCR.

### ChIP-sequencing analysis

Cells were grown to 1 (within ±0.1) × 10^7^ cells/mL in 500 mL of YES. The following procedure was identical to that of ChIP-qPCR analysis except for qPCR. In the ChIP-seq analysis of *ade6-m210* cells, DNA was recovered using the spin column-based QIAquick PCR Purification Kit (Qiagen) instead of ethanol precipitation. The ChIP libraries for the Illumina platform were prepared according to the manufacturer’s instructions. The libraries from *ade6-m210* and *otr1R*::*ade6*^+^ strains were sequenced on the Illumina HiSeq 1500 system (single-end, 51 bp) and the HiSeq 2500 system (single-end, 101 bp), respectively. The sequenced reads were mapped onto the *S*. *pombe* genome (972) using BWA (version 0.7.17), and then processed using SAMtools (version 1.6) and MACS (version 2.1.1). MACS extended each read to the expected fragment length of 200 bp using the option—extsize 200. The processed ChIP-seq data were loaded into IGV. Blue graphs indicate normalized piled-up fragment counts at single base-pair resolution: the scale of the vertical axis is represented as fragment counts per five million mapped reads. The number of both raw and mapped reads is listed in S4 Table. The experiments were not repeated.

### Western blotting analysis

Cells grown to 1 × 10^7^ cells/mL in 15 mL of YES were harvested, washed, resuspended in water, and heated at 95°C for 5 min. An equal volume of buffer containing 8 M Urea, 4% SDS, 0.12 M Tris-HCl (pH 6.8), 20% glycerol, and 0.6 M β-mercaptoethanol was added to cell suspensions, and the cells were homogenized by bead beating. The cell extracts were heated at 95°C for 5 min and spun down, and then the supernatants were recovered. The supernatant samples were separated by polyacrylamide gel electrophoresis, and the proteins were blotted onto nitrocellulose membranes. The membranes were first probed with the anti-FLAG (M2, Sigma-Aldrich) and anti-α-tubulin (B-5-1-2, Sigma-Aldrich) antibodies, and then with horseradish peroxidase-conjugated anti-Mouse IgG (GE Healthcare).

### Yeast two-hybrid assay

Matchmaker GAL4 Two-Hybrid System 3 (Clontech) was used for the Epe1-Swi6 interaction analysis. pGBKT7 (containing *TRP1*) was used for bait expression. pGADT7 (containing *LEU2*) was used for prey expression. pGBKT7 and pGADT7 plasmids were introduced into the AH109 host strain by a polyethylene glycol/lithium acetate (PEG/LiAc)-mediated method. AH109 harbors a *HIS3* reporter gene. Yeast strains were cultured on proper minimal synthetic dropout (SD) media according to the user manual (Clontech). 3-AT (15 mM; 3-amino-1,2,4-triazole), an inhibitor of the *HIS3* product, was added for precise analysis of the Epe1-Swi6 interaction. Glucose was applied to SD media as the carbon source. The experimental procedure for serial dilution assays of *S*. *cerevisiae* strains was the same as for *S*. *pombe* strains.

### Colony color assessment

Six hundred cells were spread onto a plate with a 90 mm diameter containing adenine-limited media, which generated 200–500 colonies depending on the mutation. For cell spreading, sterile glass beads were used. The cells were incubated at 30°C for 4 days on adenine-limited media, and photographed for assessment. No image processing software was applied to the assessment. Colony color on adenine-limited medium was assessed in randomized photographs and the sample names were masked. The color was grouped into five types: red or dark red; pale red; reddish pink or pink; light pink; and completely white color. The group “completely white” only included the white color observed in *ade5*Δ cells. Colonies too small for color assessment were grouped into “too small” and excluded from the percentage graph. Although the color of small colonies appeared pale, colony color was not adjusted for colony size in the assessment. When samples displayed a uniform color (at least >99%), the assessment area was reduced to half of the plate. The number of colonies of a particular color type is shown in S1 Table.

### Co-immunoprecipitation analysis

Co-immunoprecipitation analysis was performed as described previously with some modifications [12, 14]. Cells grown to 1 (within ±0.1) × 10^7^ cells/mL in 50 mL of YES were harvested by centrifugation, washed with 2 × HC buffer (200 mM HEPES-KOH (pH 7.5), 300 mM KCl, 2 mM EDTA, and 20% glycerol), frozen in liquid nitrogen, and stored at −80°C. Cells were resuspended in 2 × HC buffer containing a protease inhibitor cocktail and 2 mM DTT, and then homogenized with 16 cycles of bead beating for 15 s at 4°C to render 90% of them refraction-negative under a light microscope. The cell lysate was centrifuged for 10 min at 14,000 × *g* at 4°C, and the resultant supernatant was recovered. Twenty microliter of the supernatant was mixed with an equal volume of 2 × Laemmli buffer (8 M Urea, 2% SDS, 0.12 M Tris-HCl (pH 6.8), 20% glycerol, and 0.6 M β-mercaptoethanol) and heated at 95ºC for 5 min. Before IP, Dynabeads M-280 Sheep anti-Mouse IgG (Invitrogen) were washed once with 1 × HC buffer and incubated with 1 μg of anti-FLAG (mouse monoclonal, M2, Sigma-Aldrich) antibody for 2 h with mild rotation at 4°C followed by washing with 1 × HC buffer. The beads were suspended in the cell lysate mixed with an equal volume of 200 mM KCl containing a protease inhibitor cocktail, and incubated for 4 h with mild rotation at 4°C, where IP reaction was actually performed in 1 × HC buffer containing 250 mM KCl and 1 mM DTT. After IP, the beads were washed eight times with 1 × HC buffer containing 250 mM KCl, suspended in 20 μl of 1 × HC buffer followed by addition of an equal volume of 2 × Laemmli buffer, heated at 95ºC for 5 min, and spun down. The denatured samples were separated by polyacrylamide gel electrophoresis, and the proteins were blotted onto nitrocellulose membranes. The membranes were first probed with the anti-FLAG and anti-Swi6 antibodies, and then with horseradish peroxidase-conjugated anti-Mouse and anti-Rabbit IgG (GE Healthcare), respectively.

### Tethered transcription assay

To construct the reporter plasmid, a sequence including three Gal4-binding sites of the *GAL10* promoter was amplified with the primers 5’-CTTGCATGCGTGAAGACGAGGACGCAC-3’ and 5’-CTCATTGCTATATTGAAGTACGG-3’ from the *S*. *cerevisiae* W303 genome; the 154-bp region of the *nmt1* promoter, which contained core promoter sequence but lacked the thiamine regulatory element [43], was amplified with the primers 5’-CCGTACTTCAATATAGCAATGAGGCAGCGAAACTAAAAACCG-3’ and 5’-GTCGACATGATTTAACAAAGCGAC-3’; the coding sequence of *ade6-m210* was amplified with the primers 5’-CTTTGTTAAATCATGTCGACGAGCGAAAAACAGGTTGTAG-3’ and 5’-TTTACCCGGGCTATGCAGAATAATTTTTCCAACC-3’ from the *S*. *pombe* FY648 genome. These DNA fragments were fused by the polymerase chain reaction (PCR)-based method as previously described [66]. The fused fragment was cut with *Sph*I and *Xma*I and ligated into the pSLF173 plasmid [70] digested with the same enzymes for removal of the *nmt1* promoter, and the resultant plasmid was named pGNP154-Am. To construct expression plasmids, the 3FLAG-Gal4DBD fragment was made by PCR fusion and ligated into the pREP41 plasmid with *Nde*I and *Bam*HI. The resulting plasmid was used as an empty (control) plasmid, named pNFD41. An SV40 nuclear localization signal (NLS) sequence and a multi-cloning site (MCS) were added to the 5’ end of the forward and reverse primers, respectively, to amplify the Gal4DBD sequence (5’-GACAAGGGTGGTGGCTCCCCAAAAAAGAAGAGAAAGGTCGAAGACGCAATGAAGCTACTGTCTTCTATCG-3’ and 5’-TTCTGGATCCGTCGACGCGGCCGCCATGGAACCTCCTCCCGATACAGTCAACTGTCTTTG-3’). The extended Gal4DBD fragment was then fused to the 3FLAG fragment by another PCR to make the 3FLAG-Gal4DBD fragment. Target sequences, *epe1* mutants or the transactivation domain (TAD) of VP16 derived from human herpesvirus 1, were ligated into the empty plasmid with *Nco*I and *Sal*I. Not all primers used for construction of expression plasmids are shown because of the complexity. The plasmids are listed in S7 Table. The TP4-1D strain, harboring *ade6-M216*, was transformed with reporter and expression plasmids, which contain *ura4* and *LEU2* markers, respectively. The transformants were cultured on PMG medium lacking uracil and leucine with 15 μM of thiamine. Serial dilution assay was performed using PMG medium lacking uracil, leucine, and adenine without thiamine. Control and assay plates were incubated at 30°C for 8 days.

### Accession numbers

The sequences of the probes and the original data from the microarray experiments were deposited in GEO (http://www.ncbi.nlm.nih.gov/geo) under accession number GSE108448. The sequencing data of ChIP-seq analyses including input and IP were deposited in DDBJ (https://ddbj.nig.ac.jp/DRASearch) under accession number DRA006424 for the *ade6-m210* strains and DRA006425 for the *otr1R*::*ade6*^+^-harboring strains.

## Supporting information

S1 Fig Loss of *epe1* induces ectopic heterochromatin formation and phenotypic alterations. (A) Percentage of the colored and white colonies. Total counts are shown below the graph. The number of colonies of each color is shown in S1 Table. Colonies that were too small were excluded from the color assessment. (B) Colony color of *epe1*Δ W164–166 clones on adenine-limited (Low Ade) medium. *epe1*Δ W164–166 and *epe1*Δ W70 clones share the same parental strain. (C) ChIP-qPCR analysis of H3K9me at *otr1R*::*ade6*^+^ and *ade5* in *epe1*Δ W164–166 clones. (D) Scatter plots of transcriptome analysis comparing *epe1*Δ W70 and R69 clones and the WT strain. Log_2_ of signal intensity was plotted. Unreliable signals with low intensity were excluded. Black dot, signal changed by 1.5-fold or more; gray dot, signal changed by less than 1.5-fold; red solid line, unchanged; pink broken line, changed by 1.5-fold; navy solid line, reduced by 16-fold. Transcripts that decreased by more than 16-fold are indicated by gray numbering in the graphs and listed in the left box. Fold changes of gene expression levels of major heterochromatin assembly factors in R69 or W70 clone cells over those in WT cells are listed in the right box. N/A, not available due to low signal intensity. (E) ChIP-qPCR analysis of H3K9me at *nsa2*, *puf6*, and *SPCC569*.*03*. (F) ChIP-qPCR analysis of Swi6 at *puf6*, *SPCC569*.*03*, *cdc2*, and *otr1R*::*ade6*^+^. *cdc2*, euchromatic gene; *otr1R*::*ade6*^+^, heterochromatic marker gene. **p < 0.05 (two-tailed Student’s *t*-test). The background level in Swi6 ChIP analysis was high. (G) Ten-fold serial dilution assay for diploid strains. Control strains were spotted on adenine-limited medium. ChIP-qPCR data are represented as mean ± SD of three independent experiments (n = 3).
(TIF)

S2 Fig The red-white variegation phenotype is linked to ectopic heterochromatin formation. (A) Comparison of colony color of the strains with deletion of *ago1* and/or *taz1* with and without *epe1*Δ on adenine-limited medium. (B) Percentage of the colored and white colonies of the multiple deletion mutants shown in (A). The number of grouped colonies is shown in S1 Table. *ago1*Δ as well as *ago1*Δ *taz1*Δ induced variegated colony formation speed, resulting in formation of small colonies. The color of them looked pale, and, consequently, the pink and white groups consisted entirely of small colonies. In contrast, *epe1*Δ and *epe1*Δ *ago1*Δ strains can produce normal-size pink/white colonies (Fig 2A and 2B), suggesting different variegation mechanisms. (C) Ten-fold serial dilution assay. Some of the isolates obtained from *epe1*Δ *ago1*Δ and *epe1*Δ *taz1*Δ strains were spotted on adenine-limited medium. (D) qRT-PCR analysis of *ade1* and *ypt7* transcript levels. Data are represented as mean ± SD of three independent experiments (n = 3). **p < 0.05 (two-tailed Student’s *t*-test).
(TIF)

S3 Fig Stochastic formation of ectopic heterochromatin and development of islands constitute the diversified epigenotypes of the *epe1*Δ strain. (A) Percentage of the colored and white colonies of *epe1*Δ *ago1*Δ and *epe1*Δ *ago1*Δ W173 strains shown in Fig 3B. (B) ChIP-qPCR analysis of H3K9me at *nsa2*, *puf6*, *ade5*, and *SPCC569*.*03* in the *otr1R*::*ade6*^+^ *epe1*Δ *ago1*Δ W173 clone. (C) ChIP-sequencing analysis of H3K9me in *ade6-m210* clones. Heterochromatin island positions and *subtel3L*, *cen1L*, and *clr2* regions are shown. Data are shown as normalized fragment counts. Bar, 5 kb; open arrowhead, essential gene based on PomBase; filled arrowhead, nonessential gene. H3K9me-deposited positions are listed in S2 Table. (D) qRT-PCR analyses of *pdi4* and *can1* transcript levels. **p < 0.05 (two-tailed Student’s *t*-test). The separated data were obtained from two independent analyses. ChIP-qPCR and qRT-PCR data are represented as mean ± SD of three independent experiments (n = 3).
(TIF)

S4 Fig Epe1 prevents the ectopic heterochromatin-mediated red-white variegation via an N-terminal transcriptional activation domain-dependent mechanism. (A) Expression of 3FLAG-tagged Epe1 and its mutants. Proteins extracted from cells expressing the indicated types of FLAG-tagged Epe1 were separated by 6% polyacrylamide gel and analyzed by Western blotting with an antibody against FLAG (upper panel). WT cells were used as the no tag control. *, non-specific band. As a loading control, α-tubulin was used (lower panel). The samples subjected to the two antibodies were identical. (B) Ten-fold serial dilution assay of the ade5*-transferred strain (*ade6-m210 epe1*Δ W-t1) on adenine-limited medium. (C) ChIP-qPCR analysis of H3K9me in the ade5*-transferred strain at *nsa2*, *puf6*, *ade5*, and *SPCC569*.*03*. (D) ChIP-qPCR analyses of H3K9me2 (left) and me3 (right) in diploid cells. qPCR signals at *ade5* and *SPCC569*.*03* were monoallelic and biallelic, respectively. (E–F) ChIP-qPCR analyses of H3K9me (E) and Swi6 (F) at *IRC3*, *dg*, and *ade5*. **p < 0.05 (two-tailed Student’s *t*-test). (G) Yeast two-hybrid analysis of the *HIS3* reporter gene. Ten-fold serial dilution assay was performed. The interaction between murine p53 and SV40 large T-antigen (p53/T) was used as a common positive control. Minus TL, lacking Trp and Leu; −TLH, lacking Trp, Leu, and His; 3-AT (3-amino-1,2,4-triazole), an inhibitor of the *HIS3* product. Epe1 expressed as bait activated transcription of reporter genes without prey (the lower–TLH plate); 15 mM 3-AT masked the activation. (H) Colony color of the *bdf2*Δ strain harboring the *ade6-m210* background (left). Percentage of colored and white colonies is shown (right). (I) Yeast two-hybrid analysis of the *HIS3* reporter gene. Minus TL, lacking Trp and Leu; −TLH, lacking Trp, Leu, and His. ChIP-qPCR data are represented as mean ± SD of three independent experiments (n = 3).
(TIF)

S5 Fig JmjC-mediated incomplete suppression of ectopic heterochromatin provides metastable epigenetic variation. (A) ChIP-qPCR analysis of H3K9me at *act1*, *nsa2*, *puf6*, and *SPCC569*.*03*. (B) ChIP-qPCR analysis of H3K9me for diploid strains at *SPCC569*.*03*. The qPCR signals were biallelic. **p < 0.05 (two-tailed Student’s *t*-test). Data are represented as mean ± SD of three independent experiments (n = 3).
(TIF)

S1 Table Colony color assessment. Colony count and percentage of each color are shown.
(PDF)

S2 Table ChIP-seq peaks of *ade6-m210* strains. Peaks observed in ChIP-seq analysis of each *ade6-m210* strain are shown. Signal intensity was grouped into four types: 1, no; 2, low; 3, modest; 4, high.
(PDF)

S3 Table ChIP-seq peaks of *otr1R::ade6^+^* strains. Peaks observed in ChIP-seq analysis of each *ade6-m210* strain are shown. Signal intensity was grouped into four types: 1, no; 2, low; 3, modest; 4, high.
(PDF)

S4 Table Sequence reads. The number of raw and mapped reads in ChIP-seq data analysis is shown.
(PDF)

S5 Table Fission yeast strains used in this study. Genotypes of fission yeast strains are shown.
(PDF)

S6 Table qPCR primers used in this study. The primers used in qRT-PCR and ChIP-qPCR analyses are listed.
(PDF)

S7 Table Plasmids used for tethered transcription assay. The plasmids used in the tethered transcription analysis are listed.
(PDF)
