# Supplementary figures and images for: Regulation of ectopic heterochromatin-mediated epigenetic diversification by the JmjC family protein Epe1
Source: PLoS Genet. 2019 Jun 17;15(6):e1008129. doi: 10.1371/journal.pgen.1008129 (PMC6576747; doi:10.1371/journal.pgen.1008129)

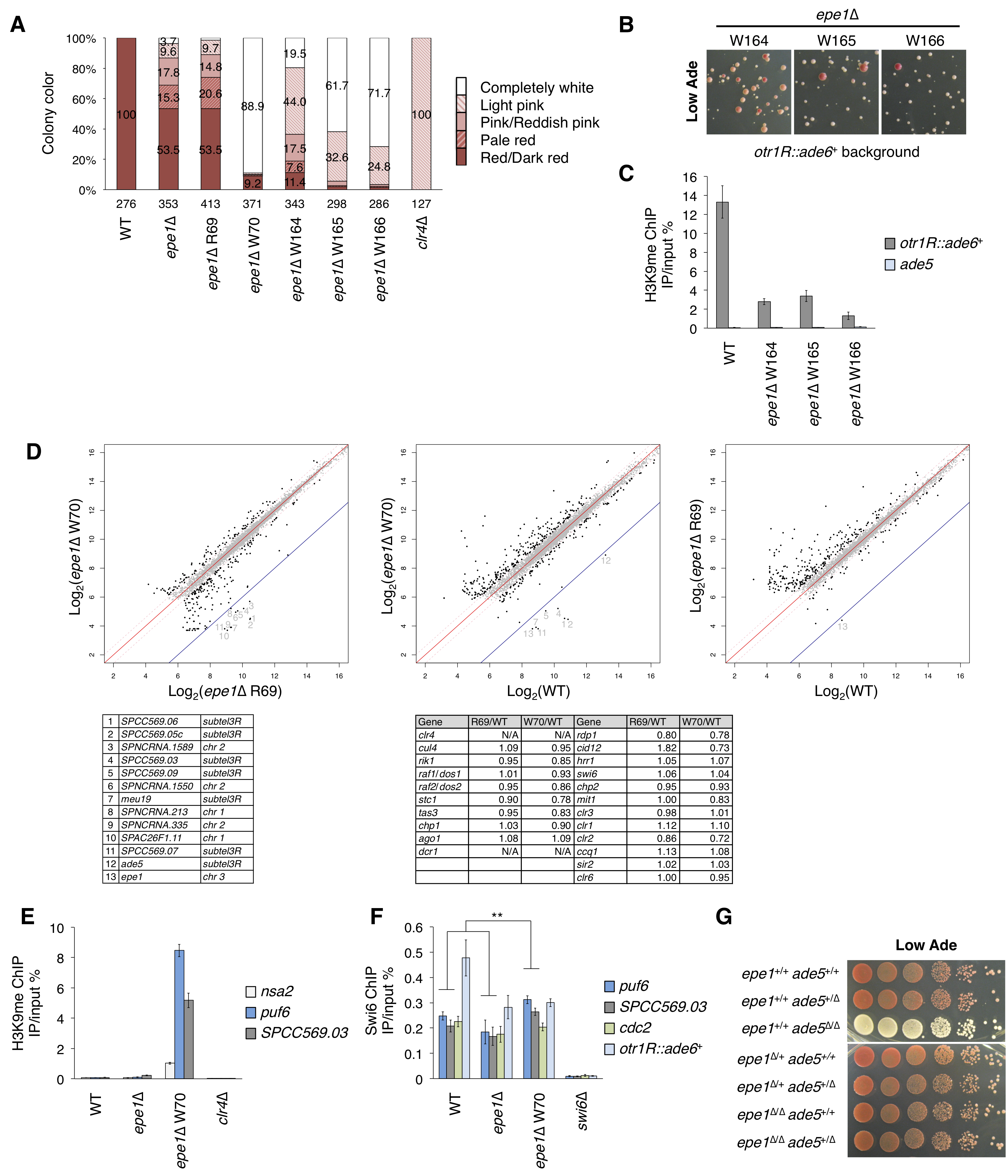

Supplement: S1 Fig — (A) Percentage of the colored and white colonies. Total counts are shown below the graph. The number of colonies of each color is shown in S1 Table. Colonies that were too small were excluded from the color assessment. (B) Colony color of epe1Δ W164–166 clones on adenine-limited (Low Ade) medium. epe1Δ W164–166 and epe1Δ W70 clones share the same parental strain. (C) ChIP-qPCR analysis of H3K9me at otr1R::ade6+ and ade5 in epe1Δ W164–166 clones. (D) Scatter plots of transcriptome analysis comparing epe1Δ W70 and R69 clones and the WT strain. Log2 of signal intensity was plotted. Unreliable signals with low intensity were excluded. Black dot, signal changed by 1.5-fold or more; gray dot, signal changed by less than 1.5-fold; red solid line, unchanged; pink broken line, changed by 1.5-fold; navy solid line, reduced by 16-fold. Transcripts that decreased by more than 16-fold are indicated by gray numbering in the graphs and listed in the left box. Fold changes of gene expression levels of major heterochromatin assembly factors in R69 or W70 clone cells over those in WT cells are listed in the right box. N/A, not available due to low signal intensity. (E) ChIP-qPCR analysis of H3K9me at nsa2, puf6, and SPCC569.03. (F) ChIP-qPCR analysis of Swi6 at puf6, SPCC569.03, cdc2, and otr1R::ade6+. cdc2, euchromatic gene; otr1R::ade6+, heterochromatic marker gene. **p < 0.05 (two-tailed Student’s t-test). The background level in Swi6 ChIP analysis was high. (G) Ten-fold serial dilution assay for diploid strains. Control strains were spotted on adenine-limited medium. ChIP-qPCR data are represented as mean ± SD of three independent experiments (n = 3). (TIF) [file pgen.1008129.s001.tif]

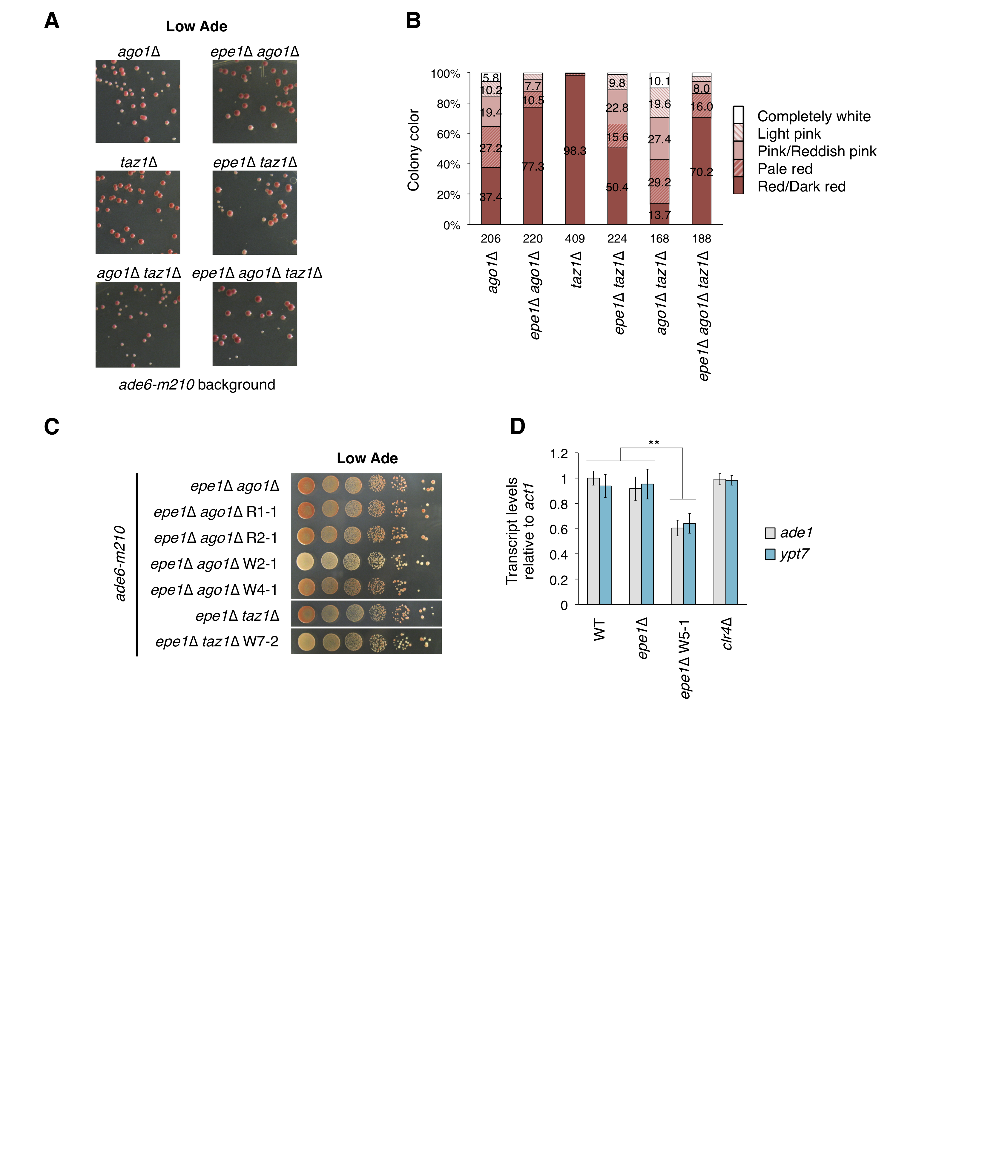

Supplement: S2 Fig — (A) Comparison of colony color of the strains with deletion of ago1 and/or taz1 with and without epe1Δ on adenine-limited medium. (B) Percentage of the colored and white colonies of the multiple deletion mutants shown in (A). The number of grouped colonies is shown in S1 Table. ago1Δ as well as ago1Δ taz1Δ induced variegated colony formation speed, resulting in formation of small colonies. The color of them looked pale, and, consequently, the pink and white groups consisted entirely of small colonies. In contrast, epe1Δ and epe1Δ ago1Δ strains can produce normal-size pink/white colonies (Fig 2A and 2B), suggesting different variegation mechanisms. (C) Ten-fold serial dilution assay. Some of the isolates obtained from epe1Δ ago1Δ and epe1Δ taz1Δ strains were spotted on adenine-limited medium. (D) qRT-PCR analysis of ade1 and ypt7 transcript levels. Data are represented as mean ± SD of three independent experiments (n = 3). **p < 0.05 (two-tailed Student’s t-test). (TIF) [file pgen.1008129.s002.tif]

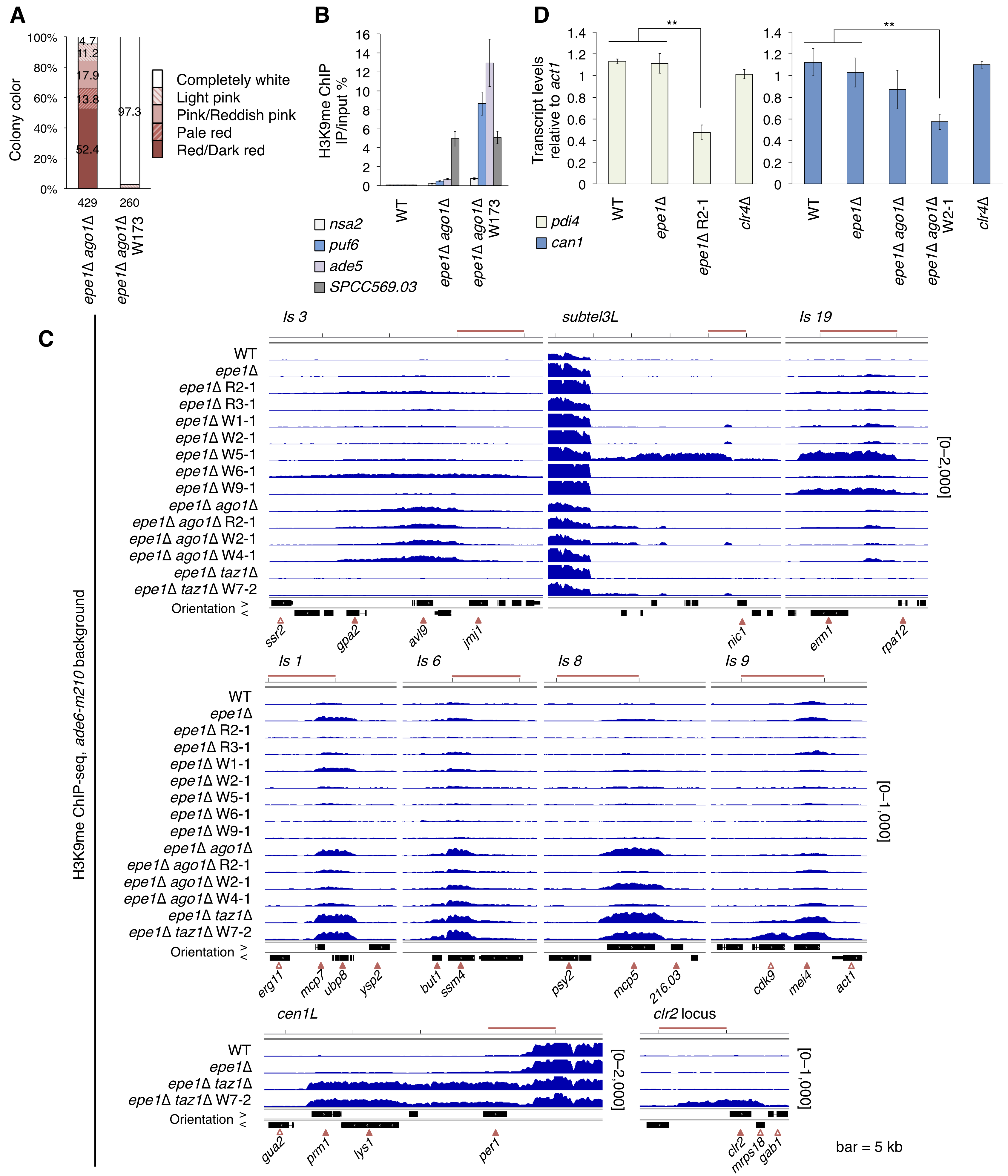

Supplement: S3 Fig — (A) Percentage of the colored and white colonies of epe1Δ ago1Δ and epe1Δ ago1Δ W173 strains shown in Fig 3B. (B) ChIP-qPCR analysis of H3K9me at nsa2, puf6, ade5, and SPCC569.03 in the otr1R::ade6+ epe1Δ ago1Δ W173 clone. (C) ChIP-sequencing analysis of H3K9me in ade6-m210 clones. Heterochromatin island positions and subtel3L, cen1L, and clr2 regions are shown. Data are shown as normalized fragment counts. Bar, 5 kb; open arrowhead, essential gene based on PomBase; filled arrowhead, nonessential gene. H3K9me-deposited positions are listed in S2 Table. (D) qRT-PCR analyses of pdi4 and can1 transcript levels. **p < 0.05 (two-tailed Student’s t-test). The separated data were obtained from two independent analyses. ChIP-qPCR and qRT-PCR data are represented as mean ± SD of three independent experiments (n = 3). (TIF) [file pgen.1008129.s003.tif]

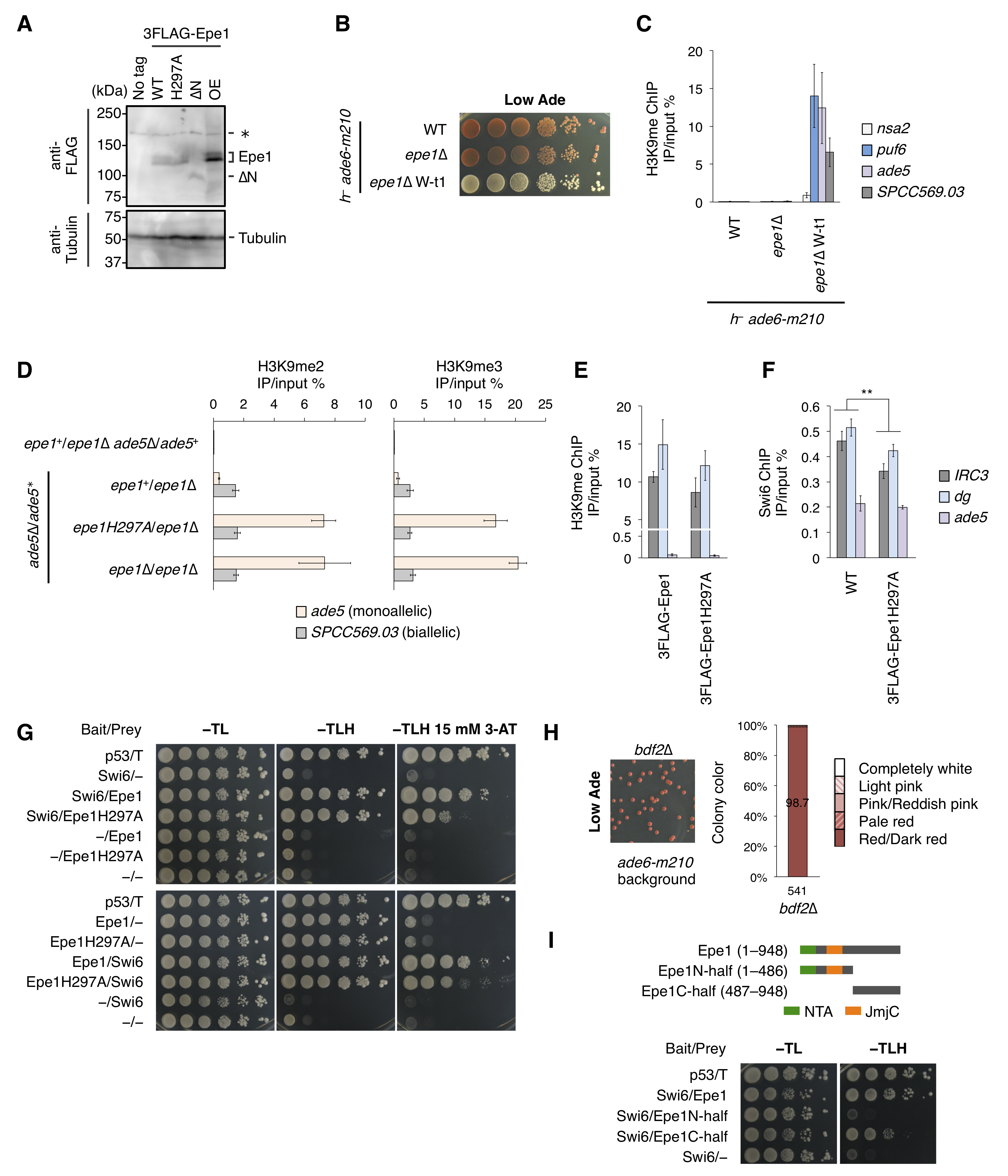

Supplement: S4 Fig — (A) Expression of 3FLAG-tagged Epe1 and its mutants. Proteins extracted from cells expressing the indicated types of FLAG-tagged Epe1 were separated by 6% polyacrylamide gel and analyzed by Western blotting with an antibody against FLAG (upper panel). WT cells were used as the no tag control. *, non-specific band. As a loading control, α-tubulin was used (lower panel). The samples subjected to the two antibodies were identical. (B) Ten-fold serial dilution assay of the ade5*-transferred strain (ade6-m210 epe1Δ W-t1) on adenine-limited medium. (C) ChIP-qPCR analysis of H3K9me in the ade5*-transferred strain at nsa2, puf6, ade5, and SPCC569.03. (D) ChIP-qPCR analyses of H3K9me2 (left) and me3 (right) in diploid cells. qPCR signals at ade5 and SPCC569.03 were monoallelic and biallelic, respectively. (E–F) ChIP-qPCR analyses of H3K9me (E) and Swi6 (F) at IRC3, dg, and ade5. **p < 0.05 (two-tailed Student’s t-test). (G) Yeast two-hybrid analysis of the HIS3 reporter gene. Ten-fold serial dilution assay was performed. The interaction between murine p53 and SV40 large T-antigen (p53/T) was used as a common positive control. Minus TL, lacking Trp and Leu; −TLH, lacking Trp, Leu, and His; 3-AT (3-amino-1,2,4-triazole), an inhibitor of the HIS3 product. Epe1 expressed as bait activated transcription of reporter genes without prey (the lower–TLH plate); 15 mM 3-AT masked the activation. (H) Colony color of the bdf2Δ strain harboring the ade6-m210 background (left). Percentage of colored and white colonies is shown (right). (I) Yeast two-hybrid analysis of the HIS3 reporter gene. Minus TL, lacking Trp and Leu; −TLH, lacking Trp, Leu, and His. ChIP-qPCR data are represented as mean ± SD of three independent experiments (n = 3). (TIF) [file pgen.1008129.s004.tif]

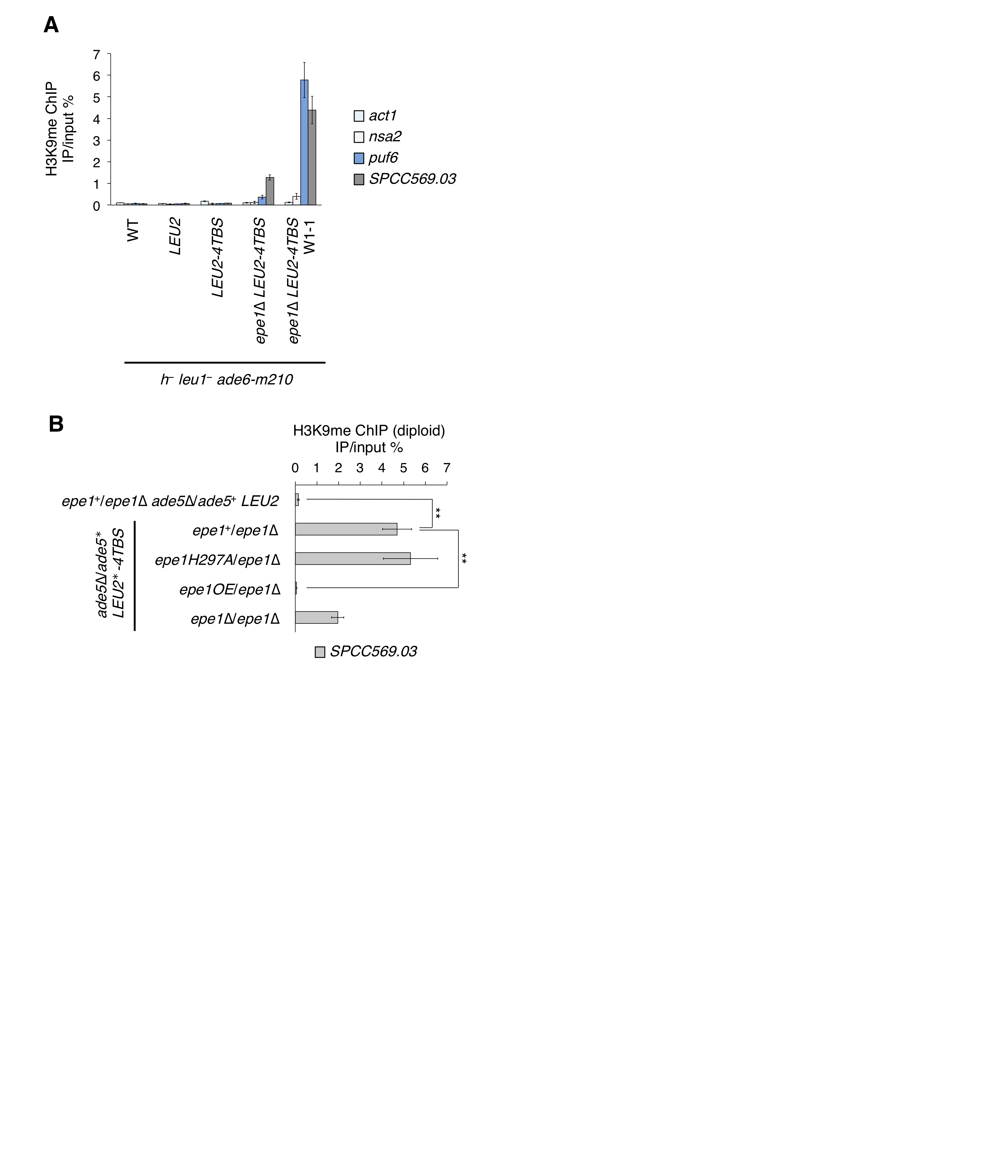

Supplement: S5 Fig — (A) ChIP-qPCR analysis of H3K9me at act1, nsa2, puf6, and SPCC569.03. (B) ChIP-qPCR analysis of H3K9me for diploid strains at SPCC569.03. The qPCR signals were biallelic. **p < 0.05 (two-tailed Student’s t-test). Data are represented as mean ± SD of three independent experiments (n = 3). (TIF) [file pgen.1008129.s005.tif]
