# Supplementary material for: Regulation of ectopic heterochromatin-mediated epigenetic diversification by the JmjC family protein Epe1
Source: PLoS Genet. 2019 Jun 17;15(6):e1008129. doi: 10.1371/journal.pgen.1008129 (PMC6576747; doi:10.1371/journal.pgen.1008129)
Supplement: S1 Table — Colony count and percentage of each color are shown. (PDF) [file pgen.1008129.s006.pdf]

Supplementary file 1. Colony color assessment

Background	Genotype	% of red/dark red	% of pale red	% of reddish pink/pink	% of light pink	% of completely white	total	red/dark red	pale red	reddish pink/pink	light pink	completely white	too small
otr1R::ade6^{+}	WT	100.0	0.0	0.0	0.0	0.0	276	276					
	*epe1*Δ	53.5	15.3	17.8	9.6	3.7	353	189	54	63	34	13	11
	*epe1*Δ R69	53.5	20.6	14.8	9.7	1.5	413	221	85	61	40	6	10
	*epe1*Δ W70	9.2	0.5	0.3	1.1	88.9	371	34	2	1	4	330	17
	*epe1*Δ W164	11.4	7.6	17.5	44.0	19.5	343	39	26	60	151	67	56
	*epe1*Δ W165	2.0	0.7	3.0	32.6	61.7	298	6	2	9	97	184	40
	*epe1*Δ W166	1.4	0.7	1.4	24.8	71.7	286	4	2	4	71	205	74
	*ago1*Δ	0.0	0.0	0.0	100.0	0.0	168				168		12
	*epe1*Δ *ago1*Δ	52.4	13.8	17.9	11.2	4.7	429	225	59	77	48	20	4
	*epe1*Δ *ago1*Δ W173	0.4	0.0	0.0	2.3	97.3	260	1			6	253	8
	*clr4*Δ	0.0	0.0	0.0	100.0	0.0	127				127		3
ade6-m210	WT	98.5	1.0	0.5	0.0	0.0	409	403	4	2			
	*epe1*Δ	61.7	11.1	13.3	11.7	2.2	316	195	35	42	37	7	10
	*clr4*Δ	96.9	2.7	0.5	0.0	0.0	413	400	11	2			2
	*clr3*Δ	99.2	0.4	0.0	0.0	0.4	254	252	1			1	
	*sir2*Δ	100.0	0.0	0.0	0.0	0.0	237	237					
	*swi6*Δ	100.0	0.0	0.0	0.0	0.0	153	153					1
	*ago1*Δ	37.4	27.2	19.4	10.2	5.8	206	77	56	40	21	12	34
	*taz1*Δ	98.3	1.2	0.5	0.0	0.0	409	402	5	2			7
	*ago1*Δ *taz1*Δ	13.7	29.2	27.4	19.6	10.1	168	23	49	46	33	17	22
	*ade5*Δ	0.0	0.0	0.0	0.0	100.0	284					284	
	3FLAG-Epe1	99.1	0.9	0.0	0.0	0.0	232	230	2				
	3FLAG-Epe1H297A	96.2	2.8	0.7	0.3	0.0	743	715	21	5	2		15
	3FLAG-Epe1ΔN	81.6	13.8	3.1	1.3	0.1	675	551	93	21	9	1	6
	*bdf2*Δ	98.7	0.7	0.6	0.0	0.0	541	534	4	3			
	*epe1*Δ *clr4*Δ	99.0	1.0	0.0	0.0	0.0	297	294	3				1
	*epe1*Δ *clr3*Δ	99.5	0.5	0.0	0.0	0.0	205	204	1				
	*epe1*Δ *sir2*Δ	100.0	0.0	0.0	0.0	0.0	210	210					
	*epe1*Δ *swi6*Δ	100.0	0.0	0.0	0.0	0.0	141	141					1
	*epe1*Δ *ago1*Δ	77.3	10.5	7.7	3.6	0.9	220	170	23	17	8	2	4
	*epe1*Δ *taz1*Δ	50.4	15.6	22.8	9.8	1.3	224	113	35	51	22	3	27
	*epe1*Δ *ago1*Δ *taz1*Δ	70.2	16.0	8.0	3.2	2.7	188	132	30	15	6	5	11
