# Supplementary material for: Regulation of ectopic heterochromatin-mediated epigenetic diversification by the JmjC family protein Epe1
Source: PLoS Genet. 2019 Jun 17;15(6):e1008129. doi: 10.1371/journal.pgen.1008129 (PMC6576747; doi:10.1371/journal.pgen.1008129)
Supplement: S2 Table — Peaks observed in ChIP-seq analysis of each ade6-m210 strain are shown. Signal intensity was grouped into four types: 1, no; 2, low; 3, modest; 4, high. (PDF) [file pgen.1008129.s007.pdf]

Supplementary file 2. ChIP-seq peaks of *ade6-m210* strains

Position	Chromosome	Gene	WT	*epe1Δ*	*epe1Δ* R2-1	*epe1Δ* R3-1	*epe1Δ* W1-1	*epe1Δ* W2-1	*epe1Δ* W5-1	*epe1Δ* W6-1	*epe1Δ* W9-1	*epe1H297A*	*epe1H297A* W2-1	*epe1Δ ago1Δ*	*epe1Δ ago1Δ* R2-1	*epe1Δ ago1Δ* W2-1	*epe1Δ ago1Δ* W4-1	*epe1Δ taz1Δ*	*epe1Δ taz1Δ* W7-2	Remarks
1	1	*SPAC977.14c*	4	3	3	1	1	2	2	3	2	4	4	3	1	2	2	3	3	*subtel1L*
2	1	*SPAC13G6.13/aps1*	1	1	1	2	1	1	1	1	1	2	1	1	1	1	1	1	1	small
3	1	*13A11.03* (*mcp7*)	2	3	2	2	3	2	2	1	1	1	2	3	2	2	2	4	4	*Is 1*
		SPAC13A11.04c (*ubp8*)	1	3	1	2	3	2	2	1	1	1	2	3	2	2	2	4	4	
4	1	*SPAC3C7.14c* (*obr1*)/*SPAC25A8.03c*	1	1	1	2	2	2	2	1	1	2	1	1	1	1	1	1	1	
5	1	*gpa2*	1	2	3	1	2	2	2	4	1	1	1	2	2	2	3	1	1	
		SPAC23H3.14 (*avl9*)	1	2	3	2	2	2	2	4	2	2	2	4	4	4	4	1	1	*Is 3*
		SPAC23H3.15c	1	2	3	2	2	2	2	4	2	2	2	4	4	4	4	1	1	
		jmj1	1	1	2	1	1	1	1	4	1	1	1	2	2	2	2	1	1	
6	1	*SPAC17A2.10c/SPAC17A2.11*	1	1	1	1	2	1	1	1	1	2	1	1	1	1	1	1	1	
7	1	*SPAC959.05c* (*pdi4*)	1	1	4	1	1	1	1	1	1	1	1	1	1	1	1	1	1	
8	1	*SPAP7G5.03* (*prm1*), *lys1*, *SPAP7G5.05* (*prl1002*)	1	1	1	1	1	1	1	1	1	1	1	1	1	1	1	4	4	*cen1L*
		SPAP7G5.06 (*per1*)	1	2	2	2	1	1	1	1	1	1	1	1	1	1	1	4	4	*cen1L*
9	1	*rad50*	1	2	1	3	1	1	1	1	1	1	1	1	1	1	1	2	2	*cen1R*
10	1	*SPAC27F1.05c/SPAC27F1.10*	1	1	1	2	2	1	1	1	1	2	1	1	1	1	1	1	1	small
11	1	*SPAC27D7.13c* (*ssm4*)	2	3	1	2	2	2	1	1	1	1	2	3	2	2	3	3	4	*Is 6*
12	1	*SPAC1B3.17* (*clr2*)	1	1	1	1	1	1	1	1	1	1	1	1	1	1	1	1	3	
13	2	*SPBPB21E7.09*	4	2	2	1	1	1	1	1	1	4	4	1	1	1	1	3	3	*subtel2L*
14	2	*mcp5*	1	2	1	1	2	2	1	1	1	1	1	3	2	3	2	4	3	*Is 8*
		SPBC216.03	1	1	1	1	1	1	1	1	1	1	1	1	1	1	1	2	2	
15	2	*SPBP35G2.10* (*mit1*)	1	1	1	1	1	1	1	1	1	1	1	1	1	1	1	1	2	
16	2	*SPBC337.02c*	1	1	1	1	1	1	2	1	1	1	3	1	2	2	1	1	2	*1
17	2	*cdk9*	1	1	1	1	1	1	1	1	1	1	1	1	1	1	1	2	3	
		mei4	2	3	1	2	2	2	2	1	1	1	2	2	2	2	2	3	3	*Is 9*
18	2	*sws1–SPBC11B10.07c* (*ivn1*)	1	1	1	1	1	1	1	1	1	1	1	1	1	1	2	1	1	
19	2	*cdk11*	1	1	1	1	1	1	1	1	1	1	1	1	1	3	1	1	1	
		can1	1	1	1	1	1	1	1	1	1	1	1	1	1	3	1	1	1	
		SPBC18H10.17c	1	1	1	1	1	1	1	1	1	1	1	1	1	3	1	1	1	
20	2	*SPBC18E5.04* (*rpl1001*)	1	1	1	1	1	1	1	1	1	1	1	1	1	1	1	3	2	
21	2	*SPBC1711.03* (*emc3*)–*SPBC1711.05* (*srp40*)	1	2	2	1	1	1	4	2	1	1	1	1	1	1	4	1	1	*mat2R*
22	2	*SPBC17G9.13c/eno101*	1	2	1	1	1	1	1	1	1	3	3	1	1	1	1	1	1	*Is 14*
23	2	*pfk1/sad1*	1	2	1	1	1	1	1	1	1	1	1	1	1	1	1	1	1	
24	2	*SPBC24C6.09c*	1	1	2	1	1	2	1	1	1	1	1	1	1	1	1	1	1	
		SPBC24C6.09c/SPBC24C6.10c	3	3	3	2	3	3	3	2	3	3	3	3	3	3	3	1	1	*Is 15*
25	2	*ade1–xdj1*	1	1	1	1	1	1	3	1	1	1	1	1	1	1	1	1	1	
26	3	*SPCP20C8.01c*	1	1	1	1	1	1	4	1	1	1	4	1	3	3	1	1	3	*subtel3L*
		SPCP20C8.01c/nic1	1	1	1	1	3	3	4	1	2	1	3	1	1	3	1	1	3	*subtel3L*
27	3	*SPCC1259.02c* (*erm1*)	1	2	2	1	2	1	4	2	4	1	1	1	2	1	1	1	1	
		SPCC1259.02c (*erm1*)/*rpa12*	1	3	3	2	3	2	4	2	4	2	2	2	3	2	3	1	1	*Is 19*
28	3	*chk1–meu27*	1	1	2	2	1	1	1	1	1	1	1	1	1	1	1	1	3	*cen3L*
29	3	*SPCC1450.09c*	1	1	1	1	1	1	1	1	1	1	1	1	1	2	1	1	1	small
30	3	*SPCP1E11.10–SPCC569.06*	1	1	1	1	4	4	1	1	4	1	4	1	1	4	1	1	4	*subtel3R*, *Is 21*
		SPCC569.03	1	1	1	1	2	2	2	1	1	1	4	1	4	4	1	1	4	*subtel3R*
		SPCC569.01c	1	1	1	1	1	1	3	1	1	1	4	1	3	3	1	1	3	*subtel3R*

*1: The *SPBC337.02c* CDS is highly homologous to *SPCC569.01c* and *SPCP20C8.01c* CDSs and is partially homomlogus to the *SPCC569.03* CDS.

Intensity	
1	No peak
2	Low
3	Modest
4	High
