# Supplementary material for: Regulation of ectopic heterochromatin-mediated epigenetic diversification by the JmjC family protein Epe1
Source: PLoS Genet. 2019 Jun 17;15(6):e1008129. doi: 10.1371/journal.pgen.1008129 (PMC6576747; doi:10.1371/journal.pgen.1008129)
Supplement: S3 Table — Peaks observed in ChIP-seq analysis of each ade6-m210 strain are shown. Signal intensity was grouped into four types: 1, no; 2, low; 3, modest; 4, high. (PDF) [file pgen.1008129.s008.pdf]

Supplementary file 3. ChIP-seq peaks of *otr1R::*$ade6^+$ strains

Position	Chromosome	Gene	WT	*epe1*Δ W70	*epe1*Δ W164	*epe1*Δ *ago1*Δ W173	Remarks
1	1	*SPAC977.06*	1	4	1	4	*subtel1L*
		SPAC977.14c	2	4	2	4	*subtel1L*
2	1	*mcp7*	1	2	4	1	*Is 1*
		ubp8	1	2	4	1	
3	1	*gpa2*	1	1	2	2	
		SPAC23H3.14 (*avl9*)	1	2	3	2	*Is 3*
		SPAC23H3.15c	1	2	3	2	
		jmj1	1	1	2	1	
4	1	*rad50*	2	2	2	1	*cen1R*
5	1	*ssm4*	1	2	2	1	*Is 6*
6	1	*SPAC144.01*	1	1	2	1	
		SPAC144.02 (*iec1*)	1	1	2	2	*Is 7*
7	1	*SPAC186.01–*	1	4	1	4	*subtel1R*
8	2	*SPBC1348.07*	1	1	4	4	*subtel2L*
		SPBPB21E7.09	1	4	2	4	*subtel2L*
9	2	*mit1*	1	2	1	1	
10	2	*SPBC337.02c*	1	2	1	2	*1
11	2	*mei4*	2	2	2	1	*Is 9*
12	2	*SPBC17G9.13*/*eno101*	1	2	2	2	*Is 14*
13	2	*pfk1*/*sad1*	1	1	2	2	
14	2	*SPBC24C6.09*/*SPBC24C6.10c*	3	3	3	3	*Is 15*
15	2	*SPBPB2B2.01–*	1	1	4	4	*subtel2R*
16	3	*SPCP20C8.01c*	1	3	1	3	*subtel3L*
		SPCP20C8.03/*SPCC1884.01*	1	2	1	2	*subtel3L*
		SPCC1884.01/*nic1*	1	3	1	3	*subtel3L*
17	3	*erm1*	1	1	3	1	
		erm1/*rpa12*	1	3	4	3	*Is 19*
18	3	*SPCP1E11.10–*	1	4	1	4	*subtel3R*, *Is 21*

*1: The *SPBC337.02c* CDS is high homologous to *SPCC569.01c* and SPCP20C8.01c CDSs and is partially homomlogus to the SPCC569.03 CDS.

Intensity

1	No peak
2	Low
3	Modest
4	High
