# Supplementary material for: Regulation of ectopic heterochromatin-mediated epigenetic diversification by the JmjC family protein Epe1
Source: PLoS Genet. 2019 Jun 17;15(6):e1008129. doi: 10.1371/journal.pgen.1008129 (PMC6576747; doi:10.1371/journal.pgen.1008129)
Supplement: S4 Table — The number of raw and mapped reads in ChIP-seq data analysis is shown. (PDF) [file pgen.1008129.s009.pdf]

Supplementary file 4. Sequence reads

Library sequencing	Strain background	Sample name	Raw reads (million)	Mapped reads (million)	Mapping efficinecy (%)
*1	*3	ChIP-seq_wildtype_648_input	10.98	10.21	93.0
		ChIP-seq_wildtype_648_H3K9me_ip	9.57	7.40	77.3
		ChIP-seq_epe1D_input	6.76	6.72	99.4
		ChIP-seq_epe1D_H3K9me_ip	9.64	7.90	82.0
		ChIP-seq_epe1D_R2-1_input	3.74	3.72	99.5
		ChIP-seq_epe1D_R2-1_H3K9me_ip	3.03	1.08	35.6
		ChIP-seq_epe1D_R3-1_input	3.46	3.44	99.6
		ChIP-seq_epe1D_R3-1_H3K9me_ip	3.16	1.23	38.8
		ChIP-seq_epe1D_W1-1_input	3.48	3.47	99.5
		ChIP-seq_epe1D_W1-1_H3K9me_ip	4.43	3.20	72.4
		ChIP-seq_epe1D_W2-1_input	3.55	3.53	99.5
		ChIP-seq_epe1D_W2-1_H3K9me_ip	3.81	2.73	71.6
		ChIP-seq_epe1D_W5-1_input	3.83	3.82	99.6
		ChIP-seq_epe1D_W5-1_H3K9me_ip	4.05	2.88	71.1
		ChIP-seq_epe1D_W6-1_input	3.98	3.95	99.5
		ChIP-seq_epe1D_W6-1_H3K9me_ip	3.67	1.03	28.0
		ChIP-seq_epe1D_W9-1_input	3.78	2.76	73.0
		ChIP-seq_epe1D_W9-1_H3K9me_ip	3.51	1.52	43.2
		ChIP-seq_epe1H297A_input	3.75	3.73	99.5
		ChIP-seq_epe1H297A_H3K9me_ip	3.64	1.51	41.4
		ChIP-seq_epe1H297A_W2-1_input	3.31	3.30	99.6
		ChIP-seq_epe1H297A_W2-1_H3K9me_ip	3.75	1.73	46.2
		ChIP-seq_epe1D_ago1D_input	3.63	3.61	99.5
		ChIP-seq_epe1D_ago1D_H3K9me_ip	3.35	2.36	70.6
		ChIP-seq_epe1D_ago1D_R2-1_input	3.92	3.90	99.5
		ChIP-seq_epe1D_ago1D_R2-1_H3K9me_ip	3.53	2.59	73.3
		ChIP-seq_epe1D_ago1D_W2-1_input	4.05	4.03	99.5
		ChIP-seq_epe1D_ago1D_W2-1_H3K9me_ip	3.88	2.45	63.0
		ChIP-seq_epe1D_ago1D_W4-1_input	3.81	3.79	99.5
		ChIP-seq_epe1D_ago1D_W4-1_H3K9me_ip	4.13	3.19	77.2
		ChIP-seq_epe1D_taz1D_input	3.39	3.38	99.5
		ChIP-seq_epe1D_taz1D_H3K9me_ip	3.26	2.72	83.5
		ChIP-seq_epe1D_taz1D_W7-2_input	3.32	3.30	99.5
		ChIP-seq_epe1D_taz1D_W7-2_H3K9me_ip	3.90	3.14	80.4
*2	*4	ChIP-seq_wildtype_2002_input	20.02	18.98	94.8
		ChIP-seq_wildtype_2002_H3K9me_ip	20.01	18.21	91.0
		ChIP-seq_epe1D_W70_input	20.01	19.47	97.3
		ChIP-seq_epe1D_W70_H3K9me_ip	20.01	17.76	88.7
		ChIP-seq_epe1D_W164_input	20.02	18.76	93.7
		ChIP-seq_epe1D_W164_H3K9me_ip	20.04	16.64	83.0
		ChIP-seq_epe1D_ago1D_W173_input	20.02	19.39	96.8
		ChIP-seq_epe1D_ago1D_W173_H3K9me_ip	20.02	17.02	85.0

*1: sequenced on Illumina HiSeq 1500 system; single-end 51 bp

*2: sequenced on Illumina HiSeq 2500 system; single-end 101 bp

*3: *ade6-m210*, no *otr1R::ade6*$^+$

*4: *otr1R::ade6*$^+$
