# Supplementary material for: Regulation of ectopic heterochromatin-mediated epigenetic diversification by the JmjC family protein Epe1
Source: PLoS Genet. 2019 Jun 17;15(6):e1008129. doi: 10.1371/journal.pgen.1008129 (PMC6576747; doi:10.1371/journal.pgen.1008129)
Supplement: S5 Table — Genotypes of fission yeast strains are shown. (PDF) [file pgen.1008129.s010.pdf]

Supplementary file 5. Fission yeast strains used in this study (continued)

Name	Genotype	Epiclone	Source
MSS422	h^+/h^- leu1-32/leu1$^+$ ade6-DN/N/ade6-DN/N ura4-DS/E/ura4-DS/E imr1L(NcoI)::ura4$^+$/imr1L(NcoI)::ura4$^+$ otr1R(SphI)::ade6$^+$/otr1R(SphI)::ade6$^+$ epe1$^+$::kanMX6/epe1$^+$::natMX6 ade5$^+$/ade5Δ::hphMX6		this study
MSS424	h^+/h^- leu1-32/leu1$^+$ ade6-DN/N/ade6-DN/N ura4-DS/E/ura4-DS/E imr1L(NcoI)::ura4$^+$/imr1L(NcoI)::ura4$^+$ otr1R(SphI)::ade6$^+$/otr1R(SphI)::ade6$^+$ epe1$^+$::kanMX6/epe1$^+$::natMX6 ade5Δ::hphMX6/ade5Δ::hphMX6		this study
MSS452	h^- leu1-32 ade6-m210 ura4-DS/E otr1R(SphI)::ura4$^+$		this study
MSS454	h^- leu1-32 ade6-m210 ura4-DS/E otr1R(SphI)::ura4$^+$ epe1Δ::natMX6		this study
MSS464	h^- leu1-32 ade6-m210 ura4-DS/E otr1R(SphI)::ura4$^+$ epe1Δ::natMX6 SPCC569.06Δ::LEU2		this study
MSS470	h^- leu1-32 ade6-m210 ura4-DS/E otr1R(SphI)::ura4$^+$ epe1Δ::natMX6 SPCC569.06Δ::LEU2-4TBS		this study
MSS473	h^+ leu1-32 ade6-m210 ura4-DS/E otr1R(SphI)::ura4$^+$ kanMX6-Purg1-3×FLAG-epe1		this study
MSS503	h^- leu1-32 ade6-m210 ura4-DS/E otr1R(SphI)::ura4$^+$ epe1Δ::natMX6	epe1Δ W-t1	this study
MSS504	h^+/h^- leu1-32/leu1-32 ade6-m210/ade6-m210 ura4-DS/E/ura4-DS/E otr1R(SphI)::ura4$^+$/otr1R(SphI)::ura4$^+$ 3×FLAG-epe1::kanMX6/epe1Δ::natMX6 ade5Δ::hphMX6/ade5$^+$	derived from epe1Δ W-t1	this study
MSS506	h^+/h^- leu1-32/leu1-32 ade6-m210/ade6-m210 ura4-DS/E/ura4-DS/E otr1R(SphI)::ura4$^+$/otr1R(SphI)::ura4$^+$ 3×FLAG-epe1H297A::kanMX6/epe1Δ::natMX6 ade5Δ::hphMX6/ade5$^+$	derived from epe1Δ W-t1	this study
MSS513	h^+/h^- leu1-32/leu1-32 ade6-m210/ade6-m210 ura4-DS/E/ura4-DS/E otr1R(SphI)::ura4$^+$/otr1R(SphI)::ura4$^+$ kanMX6-Purg1-3×FLAG-epe1/epe1Δ::natMX6 ade5Δ::hphMX6/ade5$^+$	derived from epe1Δ W-t1	this study
MSS515	h^- leu1-32 ade6-m210 ura4-DS/E otr1R(SphI)::ura4$^+$ epe1Δ::natMX6 SPCC569.06Δ::LEU2-4TBS	epe1Δ LEU2^{4TBS} W1-1	this study
MSS517	h^+/h^- leu1-32/leu1-32 ade6-m210/ade6-m210 ura4-DS/E/ura4-DS/E otr1R(SphI)::ura4$^+$/otr1R(SphI)::ura4$^+$ 3×FLAG-epe1::kanMX6/epe1Δ::natMX6 ade5Δ::hphMX6/ade5$^+$ SPCC569.06$^+$/SPCC569.06Δ::LUE2-4TBS	derived from epe1Δ LEU2^{4TBS} W1-1	this study
MSS521	h^+/h^- leu1-32/leu1-32 ade6-m210/ade6-m210 ura4-DS/E/ura4-DS/E otr1R(SphI)::ura4$^+$/otr1R(SphI)::ura4$^+$ 3×FLAG-epe1H297A::kanMX6/epe1Δ::natMX6 ade5Δ::hphMX6/ade5$^+$ SPCC569.06$^+$/SPCC569.06Δ::LUE2-4TBS	derived from epe1Δ LEU2^{4TBS} W1-1	this study
MSS529	h^+/h^- leu1-32/leu1-32 ade6-m210/ade6-m210 ura4-DS/E/ura4-DS/E otr1R(SphI)::ura4$^+$/otr1R(SphI)::ura4$^+$ kanMX6-Purg1-3×FLAG-epe1/epe1Δ::natMX6 ade5Δ::hphMX6/ade5$^+$ SPCC569.06$^+$/SPCC569.06Δ::LUE2-4TBS	derived from epe1Δ LEU2^{4TBS} W1-1	this study
MSS532	h^+/h^- leu1-32/leu1-32 ade6-m210/ade6-m210 ura4-DS/E/ura4-DS/E otr1R(SphI)::ura4$^+$/otr1R(SphI)::ura4$^+$ epe1Δ::kanMX6/epe1Δ::natMX6 SPCC569.06$^+$/SPCC569.06Δ::LUE2-4TBS	derived from epe1Δ LEU2^{4TBS} W1-1	this study
MSS533	h^+/h^- leu1-32/leu1-32 ade6-m210/ade6-m210 ura4-DS/E/ura4-DS/E otr1R(SphI)::ura4$^+$/otr1R(SphI)::ura4$^+$ epe1Δ::kanMX6/epe1Δ::natMX6 ade5Δ::hphMX6/ade5$^+$ SPCC569.06$^+$/SPCC569.06Δ::LUE2-4TBS	derived from epe1Δ LEU2^{4TBS} W1-1	this study
MSS550	h^+/h^- leu1-32/leu1-32 ade6-m210/ade6-m210 ura4-DS/E/ura4-DS/E otr1R(SphI)::ura4$^+$/otr1R(SphI)::ura4$^+$ 3×FLAG-epe1::kanMX6/epe1Δ::natMX6 ade5Δ::hphMX6/ade5$^+$ SPCC569.06$^+$/SPCC569.06Δ::LUE2		this study
MSS558	h^+/h^- leu1-32/leu1-32 ade6-m210/ade6-m210 ura4-DS/E/ura4-DS/E otr1R(SphI)::ura4$^+$/otr1R(SphI)::ura4$^+$ epe1Δ::kanMX6/epe1Δ::natMX6	derived from epe1Δ W-t1	this study
MSS562	h^+/h^- leu1-32/leu1-32 ade6-m210/ade6-m210 ura4-DS/E/ura4-DS/E otr1R(SphI)::ura4$^+$/otr1R(SphI)::ura4$^+$ epe1Δ::kanMX6/epe1Δ::natMX6 ade5Δ::hphMX6/ade5$^+$	derived from epe1Δ W-t1	this study
MSS599	h^+/h^- leu1-32/leu1-32 ade6-m210/ade6-m210 ura4-DS/E/ura4-DS/E otr1R(SphI)::ura4$^+$/otr1R(SphI)::ura4$^+$ 3×FLAG-epe1::kanMX6/epe1Δ::natMX6 ade5Δ::hphMX6/ade5$^+$		this study
MSS623	h^+ leu1-32 ade6-m210 ura4-DS/E otr1R(SphI)::ura4$^+$ 3×FLAG-epe1::kanMX6		this study
MSS624	h^+ leu1-32 ade6-m210 ura4-DS/E otr1R(SphI)::ura4$^+$ 3×FLAG-epe1H297A::kanMX6		this study
MSS650	h^- leu1-32 ade6-m210 ura4-DS/E otr1R(SphI)::ura4$^+$ SPCC569.06Δ::LEU2		this study
MSS705	h^+ leu1-32 ade6-m210 ura4-DS/E otr1R(SphI)::ura4$^+$ bdf2Δ::natMX6		this study
MSS767	h^+ leu1-32 ade6-m210 ura4-DS/E otr1R(SphI)::ura4$^+$ epe1Δ::kanMX6	epe1Δ R1-1	this study
MSS768	h^+ leu1-32 ade6-m210 ura4-DS/E otr1R(SphI)::ura4$^+$ epe1Δ::kanMX6	epe1Δ R2-1	this study
MSS769	h^+ leu1-32 ade6-m210 ura4-DS/E otr1R(SphI)::ura4$^+$ epe1Δ::kanMX6	epe1Δ R3-1	this study
MSS770	h^+ leu1-32 ade6-m210 ura4-DS/E otr1R(SphI)::ura4$^+$ epe1Δ::kanMX6	epe1Δ W1-1	this study
MSS771	h^+ leu1-32 ade6-m210 ura4-DS/E otr1R(SphI)::ura4$^+$ epe1Δ::kanMX6	epe1Δ W2-1	this study
MSS772	h^+ leu1-32 ade6-m210 ura4-DS/E otr1R(SphI)::ura4$^+$ epe1Δ::kanMX6	epe1Δ W5-1	this study
MSS773	h^+ leu1-32 ade6-m210 ura4-DS/E otr1R(SphI)::ura4$^+$ epe1Δ::kanMX6	epe1Δ W6-1	this study
MSS774	h^+ leu1-32 ade6-m210 ura4-DS/E otr1R(SphI)::ura4$^+$ epe1Δ::kanMX6	epe1Δ W8-1	this study
MSS775	h^+ leu1-32 ade6-m210 ura4-DS/E otr1R(SphI)::ura4$^+$ epe1Δ::kanMX6	epe1Δ W9-1	this study
MSS781	h^+ leu1-32 ade6-m210 ura4-DS/E otr1R(SphI)::ura4$^+$ 3×FLAG-epe1H297A::kanMX6	epe1H297A W2-1	this study
MSS795	h^+ leu1-32 ade6-m210 ura4-DS/E otr1R(SphI)::ura4$^+$ epe1Δ::kanMX6 ago1Δ::hphMX6	epe1Δ ago1Δ R1-1	this study
MSS796	h^+ leu1-32 ade6-m210 ura4-DS/E otr1R(SphI)::ura4$^+$ epe1Δ::kanMX6 ago1Δ::hphMX6	epe1Δ ago1Δ R2-1	this study
MSS797	h^+ leu1-32 ade6-m210 ura4-DS/E otr1R(SphI)::ura4$^+$ epe1Δ::kanMX6 ago1Δ::hphMX6	epe1Δ ago1Δ W2-1	this study
MSS798	h^+ leu1-32 ade6-m210 ura4-DS/E otr1R(SphI)::ura4$^+$ epe1Δ::kanMX6 ago1Δ::hphMX6	epe1Δ ago1Δ W4-1	this study
MSS805	h^+ leu1-32 ade6-m210 ura4-DS/E otr1R(SphI)::ura4$^+$ epe1Δ::kanMX6 taz1Δ::natMX6	epe1Δ taz1Δ W7-2	this study

Supplementary file 5. Fission yeast strains used in this study

Name	Genotype	Epiclone	Source
FY2002	h^{+} leu1-32 ade6-DN/N ura4-DS/E imr1L(NcoI)::ura4^{+} otr1R(SphI)::ade6^{+}		RC Allshire, *1
FY648	h^{+} leu1-32 ade6-m210 ura4-DS/E otr1R(SphI)::ura4^{+}		RC Allshire, *2
TP4-1D	h^{+} leu1-32 ade6-M210 ura4-D18 his2		M Yanagida
MSS001	h^{+} leu1-32 ade6-DN/N ura4-DS/E imr1L(NcoI)::ura4^{+} otr1R(SphI)::ade6^{+} epe1Δ::kanMX6		this study
MSS026	h^{+} leu1-32 ade6-DN/N ura4-DS/E imr1L(NcoI)::ura4^{+} otr1R(SphI)::ade6^{+} swi6Δ::hphMX6		this study
MSS069	h^{+} leu1-32 ade6-DN/N ura4-DS/E imr1L(NcoI)::ura4^{+} otr1R(SphI)::ade6^{+} epe1Δ::kanMX6	epe1Δ R69	this study
MSS070	h^{+} leu1-32 ade6-DN/N ura4-DS/E imr1L(NcoI)::ura4^{+} otr1R(SphI)::ade6^{+} epe1Δ::kanMX6	epe1Δ W70	this study
MSS075	h^{+} leu1-32 ade6-DN/N ura4-DS/E imr1L(NcoI)::ura4^{+} otr1R(SphI)::ade6^{+} clr4Δ::natMX6		this study
MSS103	h^{+} leu1-32 ade6-DN/N ura4-DS/E imr1L(NcoI)::ura4^{+} otr1R(SphI)::ade6^{+} ago1Δ::hphMX6		this study
MSS105	h^{+} leu1-32 ade6-DN/N ura4-DS/E imr1L(NcoI)::ura4^{+} otr1R(SphI)::ade6^{+} epe1Δ::kanMX6 ago1Δ::hphMX6		this study
MSS147	h^{+} leu1-32 ade6-m210 ura4-DS/E otr1R(SphI)::ura4^{+} epe1Δ::kanMX6		this study
MSS148	h^{+} leu1-32 ade6-m210 ura4-DS/E otr1R(SphI)::ura4^{+} ago1Δ::hphMX6		this study
MSS164	h^{+} leu1-32 ade6-DN/N ura4-DS/E imr1L(NcoI)::ura4^{+} otr1R(SphI)::ade6^{+} epe1Δ::kanMX6	epe1Δ W164	this study
MSS165	h^{+} leu1-32 ade6-DN/N ura4-DS/E imr1L(NcoI)::ura4^{+} otr1R(SphI)::ade6^{+} epe1Δ::kanMX6	epe1Δ W165	this study
MSS166	h^{+} leu1-32 ade6-DN/N ura4-DS/E imr1L(NcoI)::ura4^{+} otr1R(SphI)::ade6^{+} epe1Δ::kanMX6	epe1Δ W166	this study
MSS173	h^{90} leu1-32 ade6-DN/N ura4-DS/E imr1L(NcoI)::ura4^{+} otr1R(SphI)::ade6^{+} epe1Δ::kanMX6 ago1Δ::hphMX6	epe1Δ ago1Δ W173	this study, *3
MSS185	h^{+} leu1-32 ade6-DN/N ura4-DS/E imr1L(NcoI)::ura4^{+} otr1R(SphI)::ade6^{+} ade5Δ::hphMX6		this study
MSS188	h^{+} leu1-32 ade6-m210 ura4-DS/E otr1R(SphI)::ura4^{+} ade5Δ::hphMX6		this study
MSS190	h^{+} leu1-32 ade6-m210 ura4-DS/E otr1R(SphI)::ura4^{+} clr4Δ::natMX6		this study
MSS192	h^{+} leu1-32 ade6-m210 ura4-DS/E otr1R(SphI)::ura4^{+} epe1Δ::kanMX6 ago1Δ::hphMX6		this study
MSS220	h^{+} leu1-32 ade6-m210 ura4-DS/E otr1R(SphI)::ura4^{+} clr3Δ::hphMX6		this study
MSS222	h^{+} leu1-32 ade6-m210 ura4-DS/E otr1R(SphI)::ura4^{+} epe1Δ::kanMX6 clr3Δ::hphMX6		this study
MSS224	h^{+} leu1-32 ade6-m210 ura4-DS/E otr1R(SphI)::ura4^{+} epe1Δ::kanMX6 clr4Δ::natMX6		this study
MSS242	h^{+} leu1-32 ade6-m210 ura4-DS/E otr1R(SphI)::ura4^{+} sir2Δ::natMX6		this study
MSS250	h^{+} leu1-32 ade6-m210 ura4-DS/E otr1R(SphI)::ura4^{+} epe1Δ::kanMX6 sir2Δ::natMX6		this study
MSS271	h^{+} leu1-32 ade6-DN/N ura4-DS/E imr1L(NcoI)::ura4^{+} otr1R(SphI)::ade6^{+} gal1Δ::natMX6		this study
MSS279	h^{+} leu1-32 ade6-m210 ura4-DS/E otr1R(SphI)::ura4^{+} taz1Δ::natMX6		this study
MSS290	h^{+} leu1-32 ade6-m210 ura4-DS/E otr1R(SphI)::ura4^{+} epe1Δ::kanMX6 taz1Δ::natMX6		this study
MSS309	h^{+} leu1-32 ade6-m210 ura4-DS/E otr1R(SphI)::ura4^{+} swi6Δ::hphMX6		this study
MSS311	h^{+} leu1-32 ade6-m210 ura4-DS/E otr1R(SphI)::ura4^{+} epe1Δ::kanMX6 swi6Δ::hphMX6		this study
MSS332	h^{+}/h^{-} leu1-32/leu1^{+} ade6-DN/N/ade6-DN/N ura4-DS/E/ura4-DS/E imr1L(NcoI)::ura4^{+}/imr1L(NcoI)::ura4^{+} otr1R(SphI)::ade6^{+}/otr1R(SphI)::ade6^{+} epe1Δ::kanMX6/epe1Δ::natMX6	derived from epe1Δ W70	this study
MSS335	h^{+}/h^{-} leu1-32/leu1^{+} ade6-DN/N/ade6-DN/N ura4-DS/E/ura4-DS/E imr1L(NcoI)::ura4^{+}/imr1L(NcoI)::ura4^{+} otr1R(SphI)::ade6^{+}/otr1R(SphI)::ade6^{+} epe1Δ::kanMX6/epe1Δ::natMX6 ade5^{+}/ade5Δ::hphMX6	derived from epe1Δ W70	this study
MSS354	h^{+}/h^{-} leu1-32/leu1^{+} ade6-DN/N/ade6-DN/N ura4-DS/E/ura4-DS/E imr1L(NcoI)::ura4^{+}/imr1L(NcoI)::ura4^{+} otr1R(SphI)::ade6^{+}/otr1R(SphI)::ade6^{+} epe1Δ::kanMX6/epe1^{+}::natMX6	derived from epe1Δ W70	this study
MSS358	h^{+}/h^{-} leu1-32/leu1^{+} ade6-DN/N/ade6-DN/N ura4-DS/E/ura4-DS/E imr1L(NcoI)::ura4^{+}/imr1L(NcoI)::ura4^{+} otr1R(SphI)::ade6^{+}/otr1R(SphI)::ade6^{+} epe1Δ::kanMX6/epe1^{+}::natMX6 ade5^{+}/ade5Δ::hphMX6	derived from epe1Δ W70	this study
MSS365	h^{+} leu1-32 ade6-m210 ura4-DS/E otr1R(SphI)::ura4^{+} epe1Δ::kanMX6 ago1Δ::hphMX6 taz1Δ::natMX6		this study
MSS367	h^{+}/h^{-} leu1-32/leu1^{+} ade6-DN/N/ade6-DN/N ura4-DS/E/ura4-DS/E imr1L(NcoI)::ura4^{+}/imr1L(NcoI)::ura4^{+} otr1R(SphI)::ade6^{+}/otr1R(SphI)::ade6^{+} epe1Δ::kanMX6/epe1^{+}::natMX6		this study
MSS371	h^{+} leu1-32 ade6-m210 ura4-DS/E otr1R(SphI)::ura4^{+} ago1Δ::hphMX6 taz1Δ::natMX6		this study
MSS378	h^{+}/h^{-} leu1-32/leu1^{+} ade6-DN/N/ade6-DN/N ura4-DS/E/ura4-DS/E imr1L(NcoI)::ura4^{+}/imr1L(NcoI)::ura4^{+} otr1R(SphI)::ade6^{+}/otr1R(SphI)::ade6^{+} epe1^{+}::kanMX6/epe1^{+}::natMX6		this study
MSS415	h^{+}/h^{-} leu1-32/leu1^{+} ade6-DN/N/ade6-DN/N ura4-DS/E/ura4-DS/E imr1L(NcoI)::ura4^{+}/imr1L(NcoI)::ura4^{+} otr1R(SphI)::ade6^{+}/otr1R(SphI)::ade6^{+} epe1Δ::kanMX6/epe1Δ::natMX6		this study
MSS417	h^{+}/h^{-} leu1-32/leu1^{+} ade6-DN/N/ade6-DN/N ura4-DS/E/ura4-DS/E imr1L(NcoI)::ura4^{+}/imr1L(NcoI)::ura4^{+} otr1R(SphI)::ade6^{+}/otr1R(SphI)::ade6^{+} epe1Δ::kanMX6/epe1Δ::natMX6 ade5^{+}/ade5Δ::hphMX6		this study
MSS419	h^{+}/h^{-}, leu1-32/leu1^{+}, ade6-DN/N/ade6-DN/N, ura4-DS/E/ura4-DS/E, imr1L(NcoI)::ura4^{+}/imr1L(NcoI)::ura4^{+}, otr1R(SphI)::ade6^{+}/otr1R(SphI)::ade6^{+}, epe1Δ::kanMX6/epe1^{+}::natMX6, ade5^{+}/ade5Δ::hphMX6		this study

*1: [13, 28]

*2: [29]

*3: The mating type was converted to h^{90} maybe during isolation.
