# Supplementary material for: Regulation of ectopic heterochromatin-mediated epigenetic diversification by the JmjC family protein Epe1
Source: PLoS Genet. 2019 Jun 17;15(6):e1008129. doi: 10.1371/journal.pgen.1008129 (PMC6576747; doi:10.1371/journal.pgen.1008129)
Supplement: S6 Table — The primers used in qRT-PCR and ChIP-qPCR analyses are listed. (PDF) [file pgen.1008129.s011.pdf]

Supplementary file 6. qPCR primers used in this study

ID	Name	Sequence
BP13	*act1* Fw	TGCCGATCGTATGCAAAAGG
BP14	*act1* Rv	CCGCTCTCATCATACTCTTG
BP26	*ade5* Fw	GATGCTACGCTGAATGGAGT
BP27	*ade5* Rv	GCAAAGACGTTGGTATCCCA
BP28	*dg* Fw	CTGCGGTTCACCCTTAACATC
BP29	*dg* Rv	CAACTGCGGATGGAAAAAGT
BP30	*ade6* Fw	GTAGTACGCAGTTTAGACGG
BP31	*ade6* Rv	GAGCACGCTGTTGAATTGAG
SP192	*SPCC569.03* Fw	CGTTCTTGCAAGTCGATGATG
SP193	*SPCC569.03* Rv	CAAGCGGTGGGAGTTCATAG
SP214	*ade1* Fw	GTCAAGGGCTGTAAGCAAGC
SP215	*ade1* Rv	GGAGACAGCTCCAACTGAGG
SP413	*LEU2* Fw	TCGTTCCAATGTCAAGTTCG
SP414	*LEU2* Rv	CCATTTAGGACCACCCACAG
SP421	*gal1* Fw	GTGGTGGAAATGGCAGTTG
SP420	*gal1* Rv	AATGCGGCTGTTTTAGGTG
SP431	*puf6* Fw	CCAGAGCAGGATAACCACTTG
SP432	*puf6* Rv	CTTGTTTCTCAGCACCACTCC
SP433	*nsa2* Fw	CCTCAAAGCCCGACTTATACC
SP434	*nsa2* Rv	ACCACCAGATGTGACAAGACC
SP439	*IRC3* Fw	ATCTTTGGAACGCTCTTACCC
SP440	*IRC3* Rv	GAAAAACTGTGTGAGGCAACC
SP441	*can1* Fw	GGTGGCAGGAAGAAAAGAAAAG
SP442	*can1* Rv	TACGACGTGGAAAACGAATAGG
SP449	*pdi4* Fw	ATTACACCCCGTGAGTTTCG
SP450	*pdi4* Rv	CAGACTGTGGCATTTTGTCG
SP475	*ypt7* Fw	CAACCATAGGAGCGGATTTTC
SP476	*ypt7* Rv	ATAAAACGCAACCCCCAAAC
