# Supplementary material for: Regulation of ectopic heterochromatin-mediated epigenetic diversification by the JmjC family protein Epe1
Source: PLoS Genet. 2019 Jun 17;15(6):e1008129. doi: 10.1371/journal.pgen.1008129 (PMC6576747; doi:10.1371/journal.pgen.1008129)
Supplement: S7 Table — The plasmids used in the tethered transcription analysis are listed. (PDF) [file pgen.1008129.s012.pdf]

Supplementary file 7. Plasmids used for tethered transcription assay

Name	Marker
pGNP154-Am	*ura4*, Amp^R
pNFD41	*LEU2*, Amp^R
pNFD41-Epe1	*LEU2*, Amp^R
pNFD41-Epe1H297A	*LEU2*, Amp^R
pNFD41-Epe1NTA	*LEU2*, Amp^R
pNFD41-Epe1ΔN	*LEU2*, Amp^R
pNFD41-VP16TAD	*LEU2*, Amp^R
